# Chemical Bonding Trends in Y_2_ *M*_3_Si_5_ (*M* = Mn–Cu, Tc–Pd, Re–Pt): A Study within the Broad *R*_2_ *M*_3_ *X*_5_ Intermetallic Family

**DOI:** 10.1021/acs.inorgchem.5c03309

**Published:** 2025-10-13

**Authors:** Giorgio Palla, Linda S. Reitz, Riccardo Freccero, Serena De Negri, Richard Dronskowski

**Affiliations:** † Dipartimento di Chimica e Chimica Industriale, Università degli Studi di Genova, via Dodecaneso 31, 16146 Genova, Italy; ‡ Institute of Inorganic Chemistry, RWTH Aachen University, Landoltweg 1a, 52056 Aachen, Germany

## Abstract

A comparative analysis of the chemical bonding in the Y_2_ *M*_3_Si_5_ (*M* = Mn–Cu, Tc–Pd, Re–Pt) intermetallic compounds is presented, aiming at elucidating the chemical factors governing their crystallization into tetragonal (*tP*40–Sc_2_Fe_3_Si_5_), monoclinic (*mS*40–Lu_2_Co_3_Si_5_), or orthorhombic (*oI*40–U_2_Co_3_Si_5_) structures. This study provides the first comprehensive bonding investigation of Y_2_ *M*_3_Si_5_ compounds with *M* = Fe, Co, and Ni, each adopting one of the three structure types. Employing projected crystal orbital Hamilton population curves (pCOHP), integrated pCOHP (IpCOHP), and integrated crystal orbital bond index (ICOBI) analyses, the bonding scenario is revealed to be primarily dominated by polar covalent *M*–Si interactions, followed by Y–Si, with Si–Si bonds playing a secondary role. This highlights a bonding picture more complex than that predicted by the Zintl concept. Extending the analysis to all transition metals and prototypes, regardless of their thermodynamic stability, allows for a systematic comparison of bonding in both stable and metastable configurations. The covalency distribution within the unit cell, quantified as IpCOHP%, exhibits periodic trends across the transition metal series, both along periods and down groups. The maximization of *M*–Si IpCOHP% emerges as the key chemical factor in stabilizing one structure type over another, aligning with experimental observations.

## Graphical abstract

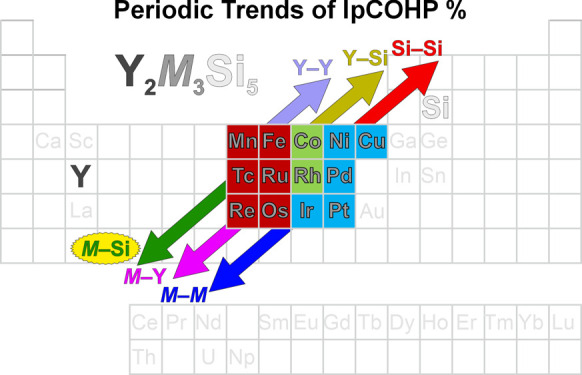

## Introduction

Intermetallic compounds constitute one of the largest classes of inorganic materials,^1^ characterized by a wide variety of crystal structures and elemental combinations, often giving rise to unique electronic structures and, consequently, distinctive physical and chemical properties.^2,3^ While the former have been extensively investigated and remain the focus of several research groups, the latter have gained significant attention only in recent years, particularly in the field of heterogeneous catalysis.^3–11^ This trend is also evident within the large *R*_2_ *M*_3_ *X*_5_ family (*R* = rare earth metal/actinide, *M* = transition metal, *X* = *p*-block element from groups 13 and 14), which comprises more than 200 known representatives crystallizing in eight different structure types.^12,13^ Superconductivity,^14,15^ Kondo behavior,^16,17^ giant magnetoresistance,^18^ and charge density wave^19^ are just a few examples of the broad range of physical properties, recently enriched by a strong potential for topological phenomena.^20^ A detailed survey on *R*_2_ *M*_3_ *X*_5_ compounds was recently published in the *Handbook on the Physics and Chemistry of Rare Earth,* ^12^ where the lack of data on their chemical bonding was highlighted. In fact, such results were available only for La_2_Pd_3_Ge_5_ (*oI*40–U_2_Co_3_Si_5_; SG: *Ibam*, No. 72),^21^ as part of our comprehensive investigation of the *R*_2_Pd_3_Ge_5_ series,^22,23^ and were further complemented by a chemical bonding analysis of La_2_Pd_3_Si_5_, the other endmember of the complete La_2_Pd_3_(Si_*x*_Ge_1–*x*_)_5_ solid solution.^24^ In both cases, the bonding analysis, primarily based on the crystal orbital Hamilton population (COHP)^25^ and its integrated value (ICOHP), revealed a much more complex scenario than predicted by a straightforward application of the Zintl formalism. Indeed, the Pd–Si­(Ge) contacts showed the highest ICOHP values, followed by the homopolar Si­(Ge)–Si­(Ge) contacts, with other previously overlooked interactions, such as La–Pd and La–Si­(Ge), making significant contributions to the overall picture. Nevertheless, these results are clearly insufficient to address a key challenge for *R*_2_ *M*_3_ *X*_5_ compounds, as pointed out by Brown et al.,^12^ understanding the relationship between structure types and their bonding. In other words, it uncovers the chemical factors that drive their crystallization into a specific structure type. The subfamily of the silicides (*X* = Si) represents a suitable playground for this type of investigation, as they adopt different structures mainly depending on the nature of the *M* metal. In particular, the *tP*40-Sc_2_Fe_3_Si_5_ structure (SG: *P*4/*mnc*, No. 128) is preferred with *M* elements from the Mn and Fe groups, whereas the *oI*40-U_2_Co_3_Si_5_ is preferred with *M* metals belonging to the Co and Ni groups. The *mS*40-Lu_2_Co_3_Si_5_ structure (SG: *C*2/*c*, No. 15) is mainly adopted by a few representatives with *M* from the Co group. This trend follows the valence electron count (VEC), as values lower than 50 lead to the Sc_2_Fe_3_Si_5_-type, while those higher than 53 typically stabilize the U_2_Co_3_Si_5_ structure. This is attributed to the fact that increasing the *d*-electron counts, which corresponds to increasing VEC, results in a gradual weakening of the *M*–*M* interactions, thereby assigning a significant role to these bonds in determining the structural preference.^12^ Nevertheless, the VEC is insufficient to fully account for the observed structural preferences, making it unsuitable for predictive purposes, thus requiring experimental confirmation, as in the case of Lu_2_Co_3_Si_5_, first reported as orthorhombic^26^ (*oI*40-U_2_Co_3_Si_5_ type) and subsequently revised as monoclinic^27^ (*mS*40-Lu_2_Co_3_Si_5_ type). Although it is reasonable to assume a polymorphic transition between the orthorhombic and monoclinic structures, given the existence of a direct group–subgroup relationship (see Figure S1), such a transition has so far been observed only for the Pr_2_Co_3_Ge_5_ germanide.^28^ These findings suggest that the two structures are stabilized by distinct bonding scenarios, despite their strong structural similarities, thus requiring in-depth investigations based on quantum–chemical methods.

Among all the *R*_2_ *M*_3_Si_5_, we selected those with *R* = Y as they have been reported with several transition metals *M*, making this series suitable for a comparative analysis. The distribution of their experimentally determined crystal structures across the different transition metals is schematically summarized in Figure 1.

**Figure 1:**
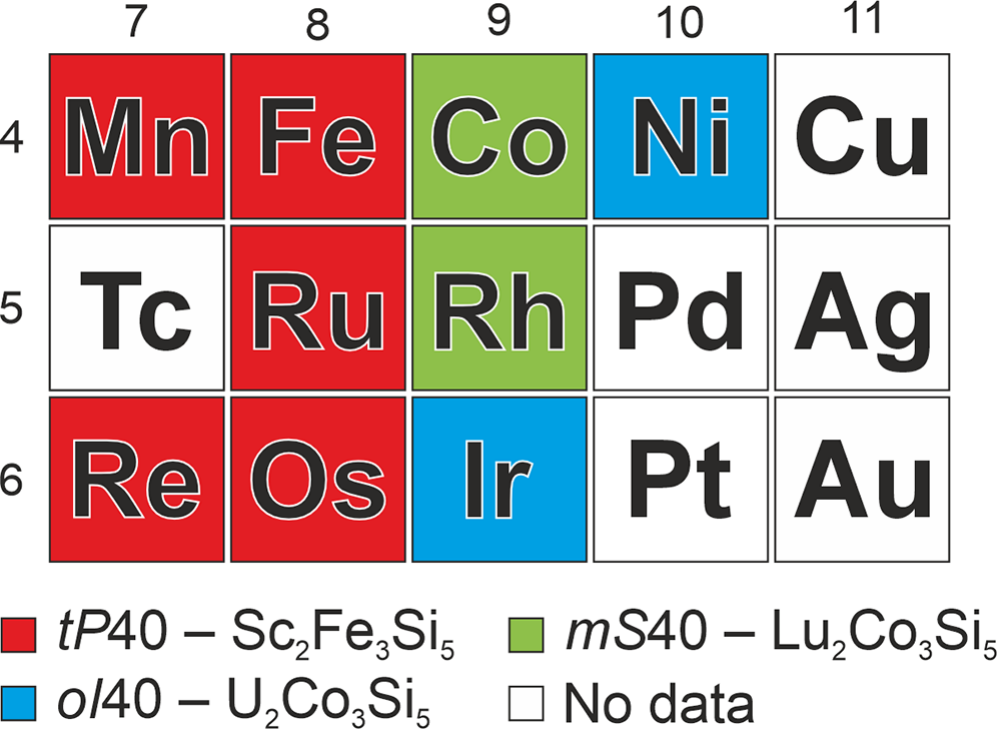
Distribution of the experimentally determined crystal structures of Y_2_ *M*_3_Si_5_ compounds depending on the nature of transition metal *M*.

The *tP*40 structure (red in Figure 1) is adopted when *M* belongs to the Mn and Fe groups,^14,29–31^ the *mS*40 is observed with Co and Rh^27,32^ (green), and the *oI*40 with Ir and Ni^33,34^ (blue). Despite Y_2_ *M*_3_Si_5_ silicides forming with many *M* elements, no compounds have been reported so far with Tc, Pd, Pt, or Cu group metals (white in Figure 1). Notably, while Pd- and Pt-containing phases are known with early lanthanides, *R*_2_Cu_3_Si_5_ has only been reported for *R* = Eu,^35^ and no *R*_2_ *M*_3_Si_5_ compounds have been obtained with *M* = Ag, Au. Therefore, in this work, we focus on the Y_2_ *M*_3_Si_5_ (*M* = Mn–Cu, Tc–Pd, Re–Pt) compounds.

In the present work, the results are presented in two separate sections. In the first, we report the outcomes of the chemical bonding analyses performed on Y_2_Fe_3_Si_5_, Y_2_Co_3_Si_5_, and Y_2_Ni_3_Si_5_, selected as representatives of the *tP*40-Sc_2_Fe_3_Si_5_, *mS*40-Lu_2_Co_3_Si_5_, and *oI*40-U_2_Co_3_Si_5_ structure types, respectively. In the second section, bonding data derived from the calculated electronic structures of all selected Y_2_ *M*_3_Si_5_ compounds (*M* = Mn–Cu, Tc–Pd, Re–Pt), each modeled in the *tP*40, *mS*40, and *oI*40 structures, are presented and analyzed to identify the chemical driving forces that govern their structural preferences.

### Computational Methods

All electronic DFT calculations were performed using the Vienna Ab-initio Simulation Package (VASP, version 5.4.4),^36–40^ with the Projector Augmented Wave (PAW) method as first described by Blöchl.^41^ The Perdew, Burke, and Ernzerhof (PBE)^42^ exchange-correlation functional within the generalized gradient approximation (GGA) was employed. The energy cutoff for the plane waves was set at 500 eV in all cases. The Brillouin zone was sampled according to the Monkhorst–Pack^43,44^ scheme, using the following *k*-point mesh for each investigated crystal structure: 10 × 8 × 14 (*oI*40), 8 × 8 × 14 (*tP*40), and 8 × 8 × 14 (*mS*40). A full structural relaxation, i.e., optimizing both atomic positions and lattice parameters, was performed for all three structural models across the compositions. The computations were considered converged when the energy differences between two iterative steps were below 10^–7^ eV for the electronic and 10^–5^ eV for the ionic relaxations. All calculations, except for Y_2_Mn_3_Si_5_, were performed without spin polarization, as they were experimentally found to be superconductors or Pauli paramagnets.^14,26,30,33,45,46^ Since Y_2_Mn_3_Si_5_ displays a ferrimagnetic ground state (*T*_N_ = 96 K),^29^ spin polarization was included; however, no significant differences in the chemical bonding results were found compared to the nonmagnetic case. Once the electronic structure of the materials was obtained, the plane-wave-based wave functions were reconstructed using the local orbital basis suite toward electronic structure reconstruction (LOBSTER, version 5.0.0)^25,47–50^ code. The projection from a delocalized plane-wave-based wave function into localized atomic orbitals was performed using the pbeVaspFit2015 ^48^ basis set, employing the recommended basis functions. Density-based effective charges (according to Bader) were derived from the VASP charge density output, as implemented in the Henkelman code,^51–54^ and Löwdin charges were calculated using LOBSTER directly from the wave function.^48^ This was combined with wave function-based bonding indicators also extracted by LOBSTER, namely the projected COHP (pCOHP),^25,47^ and the crystal orbital bond index (COBI),^55^ with their integrated values (IpCOHP, ICOBI). The threshold for including a selected interaction in the bonding analysis was determined by defining coordination polyhedra for each species based on the maximum gap method. Moreover, given the large number of different types of bonds present in the compounds, and the need to perform comparative analysis, the cumulative integrated pCOHP/cell, and their corresponding percentage values (ICOHP%) were evaluated and analyzed. This follows a well-established approach proven to be suited for conducting comparative bonding studies in intermetallic compounds.^56–60^ The pCOHP and COBI curves were visualized with the wxDragon^61^ program, and the structural models were generated by means of the VESTA^62^ software.

## Results and Discussion

### Chemical Bonding in Y_2_ *M*_3_Si_5_ (*M* = Fe, Co, Ni)

Y_2_Fe_3_Si_5_, Y_2_Co_3_Si_5_, and Y_2_Ni_3_Si_5_ were selected for in-depth chemical bonding analysis as representatives of the *tP*40-Sc_2_Fe_3_Si_5_, *mS*40-Lu_2_Co_3_Si_5_, and *oI*40-U_2_Co_3_Si_5_ structures, respectively. More details on their crystallographic data are available in the Supporting Information (Tables S1–S3). In a first approximation, the chemical bonding can be described by applying the 8–*N* rule to the silicon sublattice. Assuming a formal charge transfer from both yttrium and the *M* metals to silicon and evaluating the presence of Si–Si covalent bonds based on interatomic distances, electroneutrality is respected for Y_2_Co_3_Si_5_ and Y_2_Ni_3_Si_5_ by the following ionic formula: (Y^3+^)_2_(*M* ^2+^)_3_[(0*b*)­Si^4*–*^]­[(2*b*)­Si^2*–*^]_4_ (*b* = bonded). As shown in Figure 2b,c, (2*b*)­Si realizes infinite zigzag chains running parallel to [001]. The same does not apply to Y_2_Fe_2_Si_5_ (*tP*40), given the occurrence of both (1*b*) and (2*b*)­Si, which yield an ionic formulation comprising excess electrons: (Y^3+^)_2_(Fe^2+^)_3_[(1*b*)­Si^3*–*^]­[(2*b*)­Si^2*–*^]_4_ × 1*e* ^*–*^. Increasing the number of electrons formally transferred from iron, i.e., assuming Fe^3+^, would not help in fulfilling electroneutrality, resulting in electron deficiency. As shown in Figure 2a, (1*b*) and (2*b*) give rise to dumbbells and zigzag chains, respectively.

**Figure 2:**
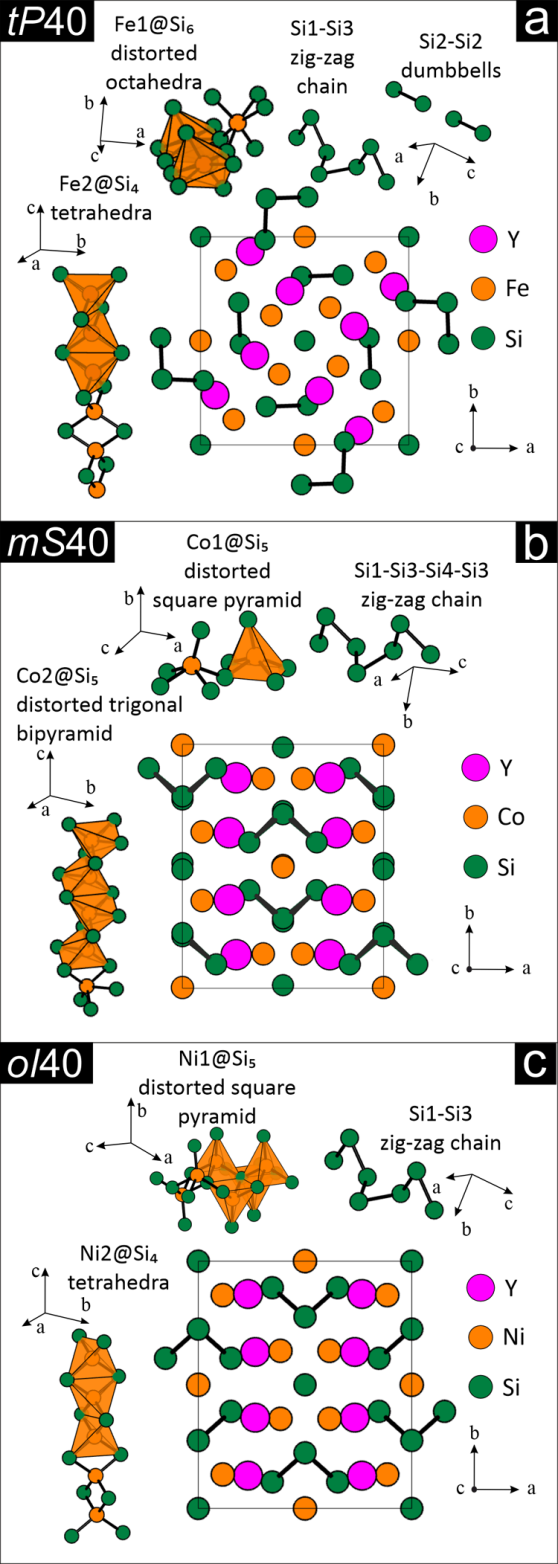
Crystal structures of (a) Y_2_Fe_3_Si_5_, (b) Y_2_Co_3_Si_5_, and (c) Y_2_Ni_3_Si_5_ selected as representative for the *t*P40-Sc_2_Fe_3_Si_5_, *m*S40-Lu_2_Co_3_Si_5_, and *oI*40-U_2_Co_3_Si_5_ types, respectively. Details on the Si-based polyanions, deduced according to interatomic distances, as well as the mutual coordination between Si and *M* are also provided.

As already pointed out for several ternary rare-earth tetrelides,^63–65^ including La_2_Pd_3_Ge_5_ and La_2_Pd_3_Si_5_,^21,24^ these formal pictures are insufficient to account for the much more complex bonding scenario, which is generally characterized by polar–covalent, rather than ionic, interactions between the cationic and anionic partial structures. To provide a more quantitative description, Table 1 presents calculated effective charges (*Q* ^eff^) according to the quantum theory of atoms in molecules (QTAIM, left) and wave function-based Löwdin charges (right), supporting this evidence (Table 1, left).

**Table 1:** QTAIM Effective Charges for Each Constituent Atom Ω of the Y_2_ *M*_3_Si_5_ (*M* = Fe, Co, Ni) Compounds and the Corresponding Löwdin Charges from the Wave Function

	*Q* ^eff^ (Ω)			Löwdin charge (Ω)		
	Y	*M*	Si	Y	*M*	Si
Y_2_Fe_3_Si_5_	+1.47	–0.37	–0.36	–0.06	+0.40	–0.22
Y_2_Co_3_Si_5_	+1.52	–0.78	–0.14	0.00	+0.42	–0.25
Y_2_Ni_3_Si_5_	+1.53	–0.98	–0.09	+0.02	+0.56	–0.34

As expected, all charges are far smaller than regular oxidation states, the usual phenomenon, because oxidation states are a rather formal issue, alluding to heteroatomic electron partitioning (on paper). Nonetheless, it is quite instructive and a bit puzzling, too, that the atomic charge assignments based either on the density (QTAIM, Bader approach) or on the wave function (Löwdin), both derived from exactly the *same* electronic ground state, arrive at different pictures, even though both cationic and anionic entities exist in both. Within QTAIM, the yttrium atom is the only cation, strongly charged (ca. +1.5e), while both transition metals and silicon appear as being anions, the transition metals even being more anionic (−0.7 ± 0.3e) than Si (−0.2 ± 0.2e). In the Löwdin representation, one finds cationic transition metals (ca. +0.46e) and anionic silicon (ca. –0.27e) in addition to practically neutral yttrium. It would be most interesting to see which of the two pictures is closer to an observable property, e.g., a chemical shift as determined by NMR. The DOS curves for all compounds show nonzero states at the Fermi level, confirming their metallic nature (Figure 3). Moreover, the Fermi level lies in a pseudogap for Y_2_Fe_3_Si_5_ and Y_2_Ni_3_Si_5_ (Figure 3a,c), deeper for the former, and in a region of lower DOS located between two relative maxima for Y_2_Co_3_Si_5_ (Figure 3b).

**Figure 3:**
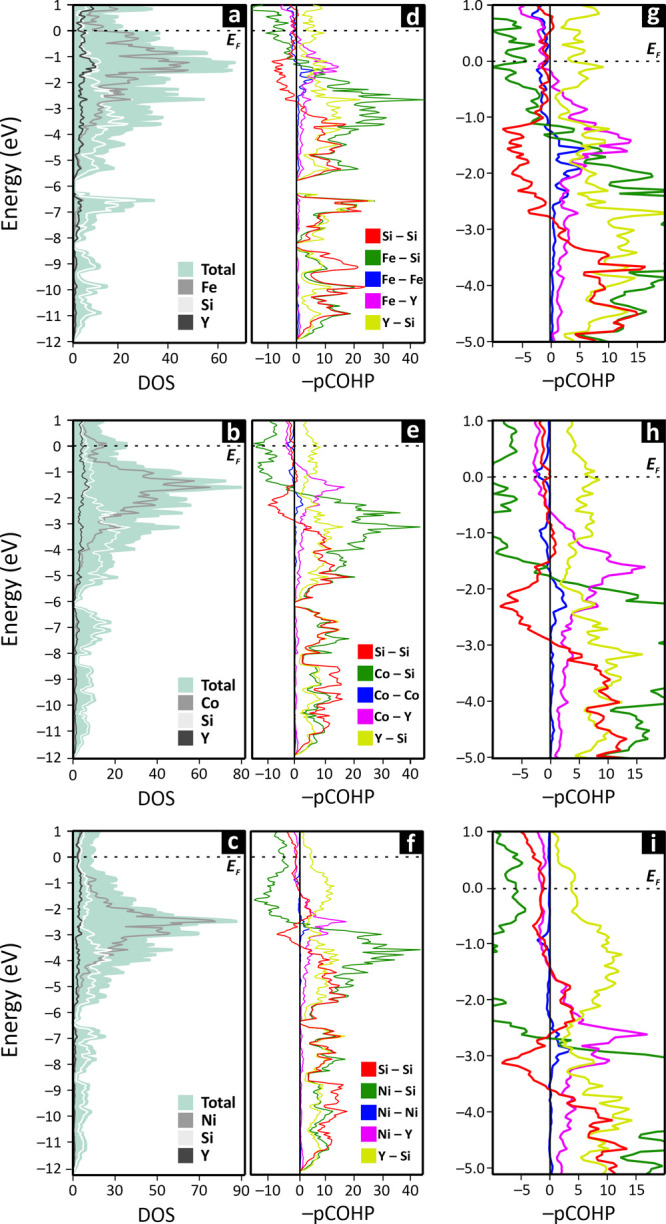
DOS (a–c) in units of (cell·eV)^−1^ and pCOHP in units of (cell)^−1^ (d–i) curves for the Y_2_Fe_3_Si_5_ (*tP*40), Y_2_Co_3_Si_5_ (*mS*40), and Y_2_Ni_3_Si_5_ (*oI*40). To enable a better view, the pCOHP curves are also reported in the range from −5 to 1 eV (g–i). The Fermi energy is set at 0 eV. The Y–Y pCOHP curves are not shown due to their low values, which hinder clear visualization.

The energy regions of the DOS dominated by the *M* states (gray lines in Figure 3a–c) correspond to their 3*d* orbital contributions. It is worth noting that as the *d*-electron count increases, the *d*-bands shift toward lower energies with a narrowing of their bandwidth, suggesting a gradual reduction in the extent of *M* involvement in covalent interactions. The Si 3*s* and 3*p* levels (light gray lines in Figure 3a–c) contribute to the region from about −12 to −6 eV, and from −6 eV to *E*_F_, respectively. The latter shows a significant energetic overlap with the 3*d M* levels, supporting the covalent nature of the Si–*M* bonds. The Y states are dispersed over the entire considered energy range (dark gray lines in Figure 3a–c) with contributions from its 4*d,* particularly in the vicinity of *E*_F_, supporting its partial ionization.

Focusing on the pCOHP curves (Figure 3d–i), it is noteworthy that despite structural differences, they all show similar trends. The pCOHP related to Si–Si interactions is predominantly bonding up to approximately −3 eV, resulting in integrated cumulative values (−IpCOHP, see Table 2) ranging from about 66 to 74 eV/cell. The values of IpCOHP per bond are listed in Table S4. This supports the covalent nature of these interactions and aligns with the previously presented formal description.

**Table 2:** Cumulative Integrated pCOHP (IpCOHP/cell) and Their Percentages to the Net Bonding Capacities (IpCOHP%/cell) for Each Type of Interaction within the Analyzed Compounds

	–IpCOHP (eV/cell)			IpCOHP%/cell		
Y_2_ *M*_3_Si_5_	Fe	Co	Ni	Fe	Co	Ni
*M*–*M*	4.55	1.67	0.91	1.35	0.53	0.29
*M*–Si	114.94	99.78	85.33	34.01	31.53	27.47
Si–Si	65.67	69.83	74.26	19.43	22.07	23.90
*M*–Y	26.67	29.11	24.19	7.89	9.20	7.79
Y–Si	121.36	112.86	123.02	35.91	35.66	39.60
Y–Y	4.77	3.19	2.96	1.41	1.01	0.95

At this point, it is worth underlining that within a Zintl framework, where covalent bonding occurs exclusively among Si atoms and ionic interactions take place between the Si substructure and the metal cations, the *M*–Si and Y–Si pCOHP curves would be expected to display narrow bands with low –IpCOHP.^47^ As is clear from both Figure 3 and Table 2, this is not the case for the systems under investigation in this article. That being said, the numerical finding does not question the simplistic Zintl picture but emphasizes its model-like concept.

The *M*–Si curves (Figure 3g–i, dark green) switch from bonding to antibonding near *E*_F_. The energy at which these transitions occur gradually decreases from Fe (−1.0 eV) to Ni (−2.5 eV), correlated with the increasing *d*-electron count. It may also be noted that this bonding–antibonding crossover coincides with the peaks in the pCOHP curves of the *M*–Y interactions (Figure 3g–i, purple) and in the DOS, corresponding to the half-filling of the *d* shell. The pCOHP of the Y–Si curves, contrary to the *M*–Si ones, are bonding up to *E*_F_ and above (Figure 3g–i, light green), clearly indicating the active involvement of Y in the chemical bonding, a feature also observed in other rare-earth ternary intermetallics.^21,24,66,67^ Moreover, the Y–Si curves are the only ones featuring significant bonding contributions at *E*_F_, compensating for the antibonding *M*–Si ones. Interestingly, the −IpCOHP for the Y–Si displays the largest values (>113 eV/cell), while the *M*–Si, which is second in the ranking of −IpCOHP, decreases from about 115 to 85 eV/cell with increasing the number of *d* electrons (−IpCOHP­(*M*–Si): *M* = Fe > Co > Ni). The Y–*M*, *M*–*M,* and Y–Y interactions contribute considerably less to the overall bonding scenario. Nevertheless, although weak, Y–*M* and Fe–Fe support covalent interactions. To give additional insights, this bonding study is further complemented by the analysis of the ICOBI values (Figure 4), which enables a more accurate description of the nature of each interaction in terms of bond order. Note that ICOBI is the periodic equivalent of the Wiberg–Mayer bond order derived from the wave function, 1 for H–H, 1.5 for an aromatic C–C bond, 2 for O=O, and 3 for N≡N. Fractional bond orders (just like for benzene) are normal in intermetallic phases, the reason being the typical undersupply of electrons in metallic systems.

**Figure 4:**
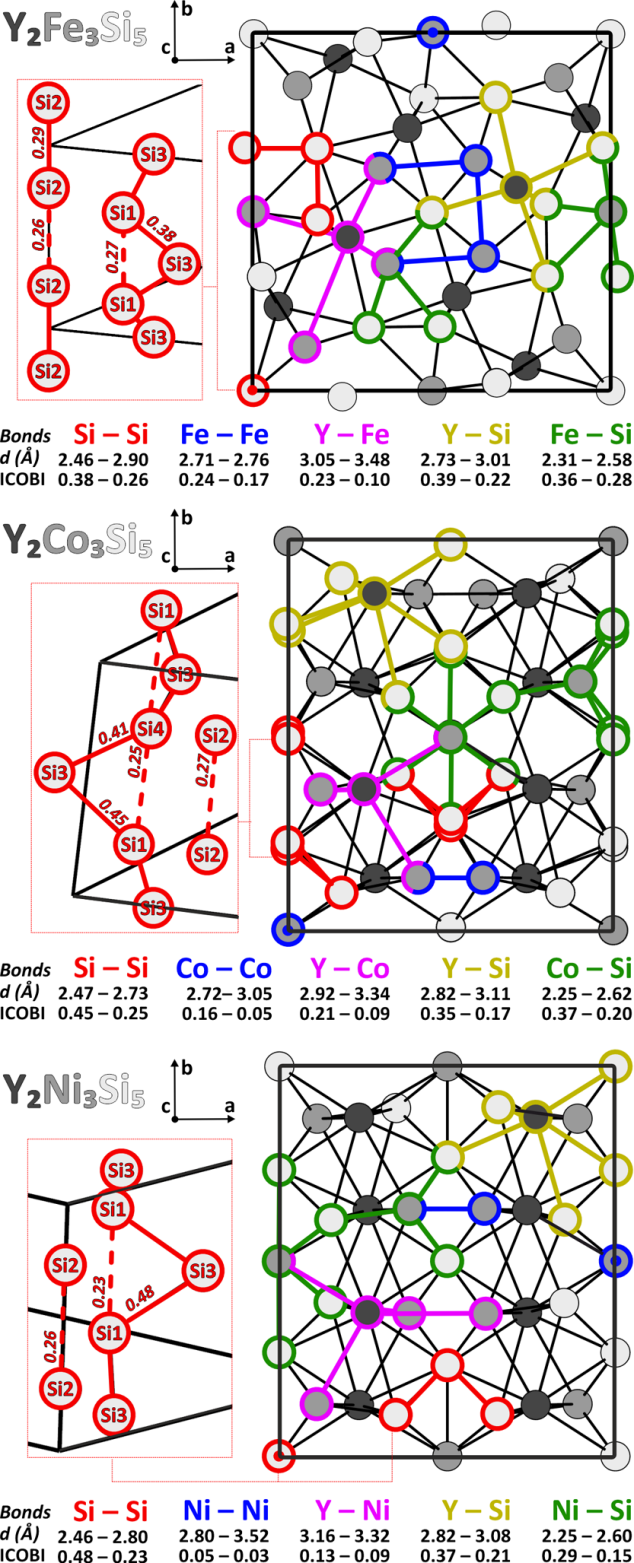
Crystal structures of the three studied compounds, viewed along the *c*-axis. Colored sticks indicate different types of contacts, for which both the distance ranges *d*(Å) and the corresponding ICOBI value ranges are reported. On the left side of each structure the Si polyanionic networks are shown, along with their ICOBI values. Dashed red lines indicate contacts that are not interpreted as covalent bonds according to the Zintl model.

For all structures, the largest ICOBI are related to all interactions, including silicon, i.e., Si–Si, Y–Si, and *M*–Si. The ICOBI for Y–Si and *M*–Si exceed the values expected for ionic interactions^55^ also at quite long distances, e.g., for Y_2_Fe_3_Si_5_ ICOBI_Y–Si_(3.01 Å) = 0.22 and ICOBI_Fe–Si_(2.58 Å) = 0.28, supporting the covalent character of these heteroatomic polar covalent interactions. At this point, it is worth focusing on the ICOBI for the Si–Si contacts. In all structures, the highest ICOBI values correspond to the shortest Si–Si distances, supporting the previously discussed partial structures: (2*b*)Si zigzag chains (ICOBI­(*tP*40)_Si1–Si3_^2.46 Å^ = 0.38, ICOBI­(*mS*40)_Si3–Si4_^2.47Å^ = 0.41, and ICOBI­(*mS*40)_Si1–Si3_^2.49 Å^ = 0.45, ICOBI­(*oI*40)_Si1–Si3_^2.46 Å^ = 0.48) and (1*b*)Si dumbbells in the *tP*40 modification (ICOBI­(*mS*40)_Si2–Si2_^2.52 Å^ = 0.29). These bonds were previously introduced in Figure 2 and are presented again using solid red sticks in Figure 4 (additional information is available in Table S4). It is worth noting that these ICOBI_Si–Si_ values are smaller than those previously reported for other ternary rare-earth silicides, i.e., ICOBI_Si–Si_ of 0.72 and 0.66 in Y_2_LiSi_2_ and Sc_2_AlSi_2_.^68^ The obtained ICOBI for longer Si–Si contacts (*d* > 2.52 Å, see Table S4), indicated by dashed red sticks in Figure 4, reveals a non-negligible covalent character. Therefore, (1*b*)Si dumbbells and isolated (0*b*)Si atoms build up infinite linear chains parallel to the *c*-axis (ICOBI­(*tP*40)_Si2–Si2_^2.90 Å^ = 0.26, ICOBI­(*mS*40)_Si2–Si2_^2.74 Å^ = 0.27, ICOBI­(*oI*40)_Si2–Si2_^2.80 Å^ = 0.26), while the zigzag fragments may be viewed as chains of silicon triangles sharing two vertices (ICOBI­(*tP*40)_Si1–Si1_^2.71 Å^ = 0.27, ICOBI­(*mS*40)_Si1–Si4_^2.73 Å^ = 0.25, ICOBI­(*oI*40)_Si1–Si1_^2.80 Å^ = 0.23). To provide a comprehensive description of the bonding scenario, interactions among the metal species must also be considered, as previously demonstrated for other ternary intermetallic tetrelides, particularly those involving transition metals and rare-earth elements. ICOBI_Y–*M*_ are equal to 0.21 and 0.23 for the shortest Y–Co and Y–Fe contacts, respectively, and decrease to 0.13 for Y–Ni (see Figure 4). This lowered ICOBI value for *M* metals with increasing valence electron count is even more pronounced for the *M*–*M* interactions, decreasing from ICOBI_Fe–Fe_(2.71 Å) = 0.24 to ICOBI_Ni–Ni_(2.80 Å) = 0.05. This trend reveals a declining tendency of the *M* metals to covalently interact, both among themselves and with Y, following the order: Fe > Co > Ni.

At this point, it is worth providing some additional comments on the ICOBI values for the interactions under consideration. Although these values indicate a significant covalent character of the bonds, they never reach a bond order of 1.0, which would typically be expected for homopolar bonds, e.g., ICOBI_C–C_ for diamond.^55^ This behavior can be ascribed both to ionic contributions, making several polar covalent interactions, and to the delocalized nature of the bonding in metallic compounds. To support this interpretation, several three-center ICOBI^(3)^ were calculated for different atomic triads (see Table S5).^66^ The results, which range from approximately 0.046 to −0.031, indeed indicate a tendency toward delocalized bonding, even though the effect is rather small and certainly less decisive than the regular two-center interactions.

Having discussed the nature of chemical bonding in (*tP*40)­Y_2_Fe_3_Si_5_, (*mS*40)­Y_2_Co_3_Si_5_, and (*oI*40)­Y_2_Ni_3_Si_5_, it is of interest to extend the analysis to the other Y_2_ *M*_3_Si_5_ compounds (*M* = Mn, Cu, Tc–Pd, Re–Pt), with particular attention devoted to identifying the key chemical factors that influence crystallization into a specific structure type depending on the *M* metal. To this aim, IpCOHP% proved particularly effective and suitable for identifying trends as a function of both the *M* metal and the crystal structure. As an example, the corresponding results for the previously analyzed phases (*M* = Fe, Co, Ni) are reported in Table 2. The trends observed, including also the other *M* metals, are discussed in the following section.

### Extension of the Bonding Analysis to the Y_2_ *M*_3_Si_5_ (*M* = Mn–Cu, Tc–Pd, Re–Pt) Series

Total energies obtained after structural relaxations for the Y_2_ *M*_3_Si_5_ compounds (*M* = Mn–Cu, Tc–Pd, Re–Pt) show that the *tP*40 structure is the most stable for Mn and Fe groups, *mS*40 for the Co group, and *oI*40 for the Ni group and Cu. Interestingly, for *M* = Mn–Ni, Ru, Rh, and Re–Ir, the lowest energy structures agree with those experimentally found (see Figure 1). While no information is available on the existence of Y_2_ *M*_3_Si_5_ with *M* = Tc, Pt, and Cu, recent investigations performed in our group reveal that Y_2_Pd_3_Si_5_ does not form.^69^ The (*mS*40)­Y_2_ *M*_3_Si_5_ structure is retained upon relaxation only for *M* = Os, Co, and Rh, as in the other cases, it transforms into the *oI*40 type, a transition facilitated by the direct group–subgroup relationship between the two structures (see Figure S1).^27^

However, to highlight trends as a function of both the crystal structure and *M*, quantum–chemical calculations and analyses were performed for all obtained structures, including those not thermodynamically stable (Table S4). As anticipated, IpCOHP% proved particularly suitable for this purpose.

The periodic nature of the IpCOHP% for each type of interaction, across both groups and periods, is clearly visible in Figures 5 and S2.

**Figure 5:**
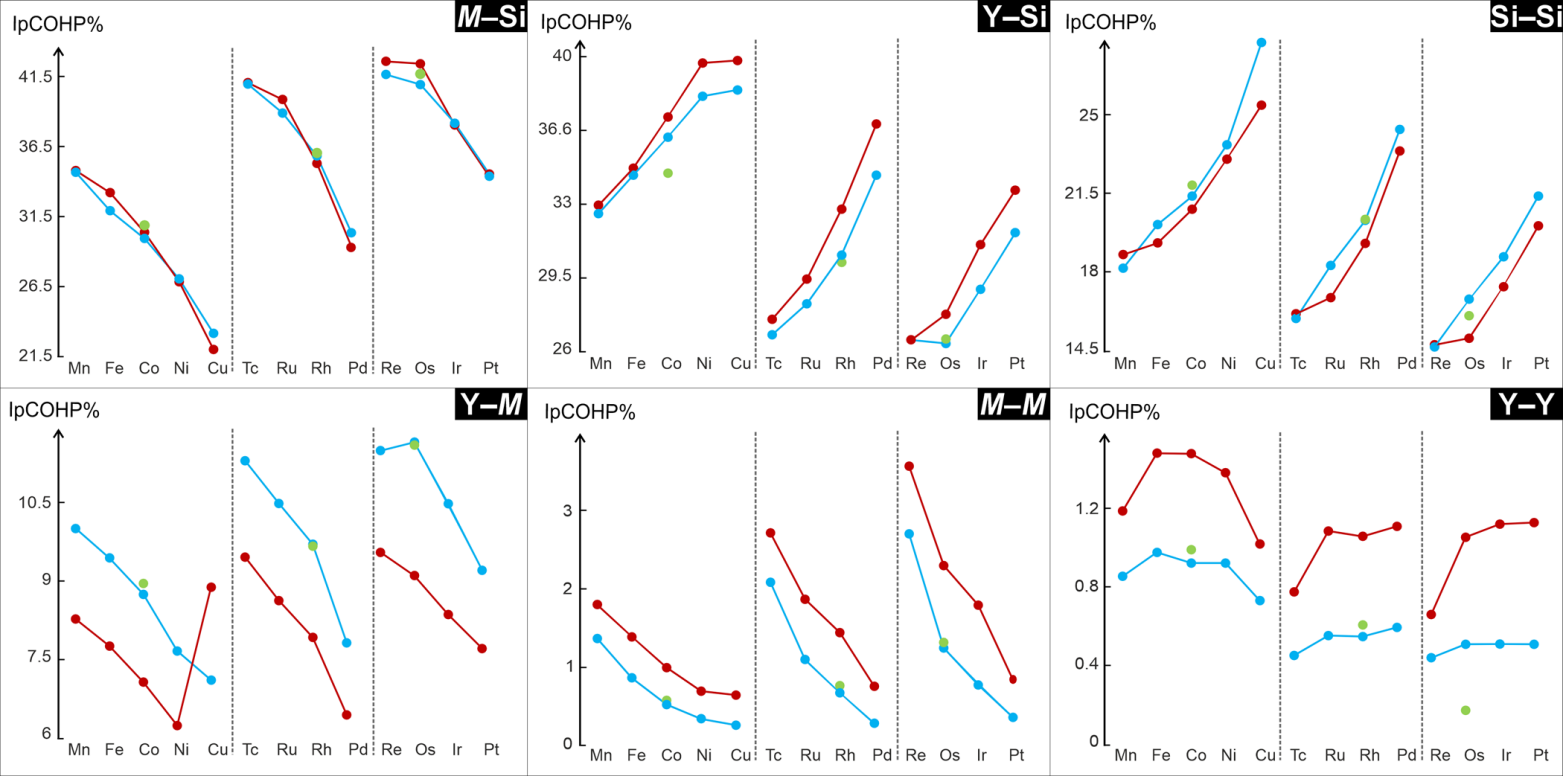
IpCOHP% vs transition metal (*M*), ordered by period. The type of interaction considered is indicated in the top-right corner of each panel. Dark red, light blue, and green dots correspond to the data for the *t* *P*40, *o* *I*40, and *m* *S*40 structure types, respectively. The segmented lines serve as eye guides to highlight trends as *M* varies.

The largest IpCOHP% values are associated with the *M*–Si, Y–Si, and Si–Si interactions, with the first two always exceeding 20%, and the latter remaining above approximately 15%. These are followed by IpCOHP%(Y–*M*) with values ranging between about 11 and 6%. The lowest contributions to the overall bonding capacities come from the homoatomic metal–metal interactions, i.e., *M*–*M* and Y–Y, with the latter falling below 2%.

While IpCOHP%(Y–Y) does not exhibit significant trends, despite a slight decrease within the group, these interactions can be neglected due to their particularly low contributions. The IpCOHP%(*M*–*M*) is always larger for the *tP*40 modification, due to the more covalent contribution of the *M*1–*M*1 (Table S4) bonds in this structure. Moreover, they clearly decrease along the period due to the gradual filling of the *d* orbitals and the resulting *d*–*d* electronic repulsion. Unlike the *M*–*M* interactions, the Y–*M* IpCOHP% values are higher in the *oI*40 and *mS*40 structures than in the *tP*40, although they also decrease across the period. This trend supports previous findings (Figure 3g–i, purple): the variation in the number of *M* electrons influences the filling of bonding and antibonding states, and consequently, the strength of the Y–*M* bond.

The IpCOHP%(Y–Si) increases along the period and decreases down the group (Figures 5 and S2), despite the nondirect participation of the transition element in these interactions. Here, a different number of valence electrons with a smaller atomic radius of the *M* elements stabilizes the interactions between yttrium and silicon (Figure S3).

The Si–Si and *M*–Si IpCOHP% display opposite trends (Figure 5): while the former increases along the period, the latter decreases. The *M*–Si pCOHP curves show antibonding states above *E*_F_ that are gradually filled as the number of *M* valence electrons increases, thereby weakening the *M*–Si interactions. At the same time, Si becomes less prone to delocalize its electrons toward *M*, making these electrons more available to stabilize Si–Si bonds.

An interesting conclusion that can be drawn from these trends is that chemical bonding in the Y_2_ *M*_3_Si_5_ compounds is more strongly influenced by the nature of the constituent elements than by the adopted crystal structure. This is evidenced by the larger variations in IpCOHP% observed when changing the transition metal *M*, compared to those resulting from structural modifications.

In this context, it is worth noting that IpCOHP%(*M*–Si) are the only quantities displaying significant trend variations as a function of both the transition metal *M* and the crystal structure. Therefore, a correlation between IpCOHP%(*M*–Si) values and the structural preference for a given composition has been identified, as illustrated in Figure 6.

**Figure 6:**
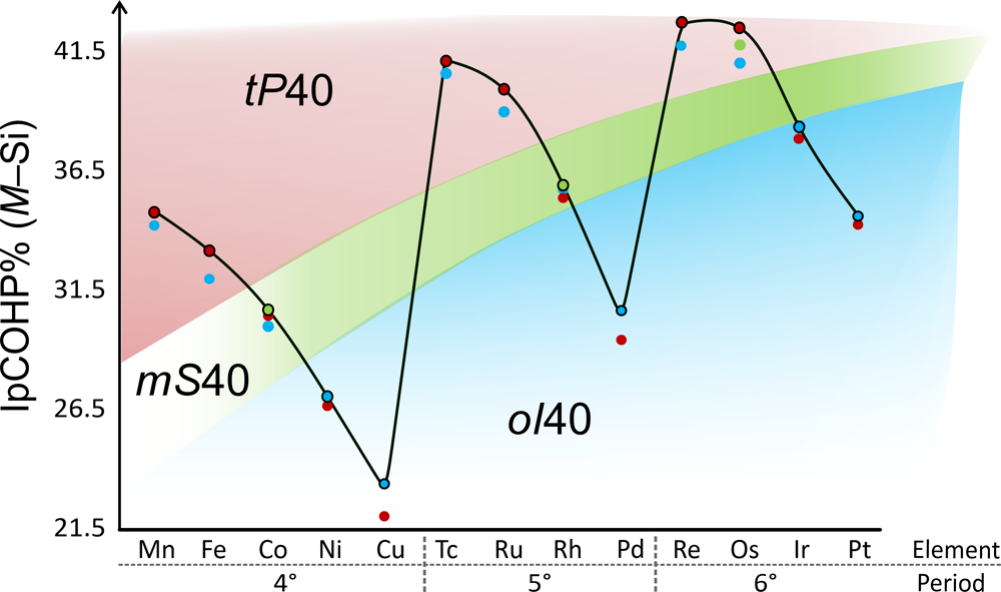
IpCOHP% vs transition metal (*M*), ordered by periods, for the *M*–Si interactions within each considered crystal structure. Red, green, and blue circles indicate data corresponding to the *t* *P*40, *m* *S*40, and *o* *I*40 structure types, respectively. The black line serves as a visual guide connecting the points with the highest IpCOHP% values for each *M* element. The shaded areas highlight the most stable crystal structure identified for each *M*.

In particular, the crystal structures identified as the most stable based on total energy calculations correspond to those exhibiting the highest IpCOHP% values for the *M*–Si bonds. This trend is illustrated in Figure 6 and is clearly visible by following the black line, which connects the highest IpCOHP% values for each *M*. It is worth reminding ourselves that total energy predictions are always consistent with experimental data, when available. This finding suggests that *M*–Si interactions play a dominant role in determining the crystallization into a specific structure type depending on the *M* metal.

## Conclusions

In this work, a quantum–chemical approach was employed to investigate the chemical bonding for the Y_2_ *M*_3_Si_5_ (*M* = Mn–Cu, Tc–Pd, and Re–Pt) series, belonging to the broader *R*_2_ *M*_3_ *X*_5_ family of intermetallic compounds. This study provides the first detailed bonding description for Y_2_ *M*_3_Si_5_ compounds (*M* = Fe, Co, Ni) crystallizing in three distinct but symmetry-related structure types. Through the combined use of −pCOHP curves, IpCOHP and ICOBI values, and Löwdin/QTAIM charges, the complex and mixed nature of the numerous bonding interactions was revealed. In particular, although the presence of covalent Si–Si bonds was confirmed, these were not found to be predominant, emphasizing the limitations of applying a Zintl-type formalism to these systems. Instead, the bonding scenario is dominated by polar covalent *M*–Si interactions, followed by the Y–Si, along with evidence of rather weak delocalized bonding from ICOBI^(3)^ values. Additionally, non-negligible covalent character was also identified for the shortest Y–*M* and *M*–*M* contacts. The analysis was extended to the full Y_2_ *M*_3_Si_5_ (*M* = Mn–Cu, Tc–Pd, Re–Pt) series by simulating each composition in all three structure types regardless of their thermodynamic stability. This allowed for a comparative bonding analysis between stable and unstable configurations, enabling the identification of structure–bonding relationships. This has been achieved by comparing the IpCOHP% values for each type of interaction, highlighting their periodic trends across the transition metal series. The key finding of this analysis is that for each composition, the most stable crystal structure corresponds to the one exhibiting the highest IpCOHP% for the *M*–Si bonds, highlighting the dominant role of *M*–Si interactions. Finally, the chemical bonding investigations performed in this study will serve as a basis for future investigations of other *R*_*2*_ *M*_*3*_ *X*_*5*_ compounds, particularly those involving different rare-earth or tetrel elements, particularly where different structures occur within the same series or where CDW-like transitions are encountered. These insights will contribute to deepening our understanding of the broader connection between the established bonding scenarios and both the chemical and physical properties of these materials.

## Supplementary Material



## Notes

Data on all investigated R_2_ *M*_3_Si_5_ silicides including experimentally determined atomic positions, a Bärninghausen tree, bonding data comprising ICOHP and three-center ICOBI^(3)^ values, IpCOHP% vs transition metal plots as well as COHP plots for the Y–Si interactions in Y_2_ *M*_3_Si_5_ (PDF)

## References

[1] Nesper R. (1991). Bonding Patterns
in Intermetallic Compounds. Angew. Chem., Int.
Ed..

[2] Amon A., Svanidze E., Ormeci A., König M., Kasinathan D., Takegami D., Prots Y., Liao Y.-F., Tsuei K.-D., Tjeng L. H., Leithe-Jasper A., Grin Y. (2019). Interplay of Atomic Interactions in the Intermetallic Semiconductor
Be_5_Pt. Angew. Chem., Int. Ed..

[3] Armbrüster M. (2020). Intermetallic
Compounds in Catalysis–a Versatile Class of Materials Meets
Interesting Challenges. Sci. Technol. Adv. Mater..

[4] Freccero R., Spennati E., Garbarino G., Riani P. (2024). Intermetallic Based
Materials for Sabatier Reaction: Surface Understanding, Performance
Assessment and Comparison with Commercial Catalyst. Applied Catalysis B: Environmental.

[5] Zhong Y., Chen X., Liu S., Liu H., Hu D., Lafaye G., Liang C. (2025). Intermetallic *R*Ni_2_Si_2_ (*R* = Ca,
La, and Y) Catalysts
with Electron-Rich Ni Sites for Continuous Flow Selective Hydrogenation
of Maleic Anhydride. ACS Appl. Mater. Interfaces.

[6] Liu S., Gong Y., Yang X., Zhang N., Liu H., Liang C., Chen X. (2023). Acid-Durable Intermetallic CaNi_2_Si_2_ Catalyst
with Electron-Rich Ni Sites for Aqueous
Phase Hydrogenation of Unsaturated Organic Anhydrides/Acids. Chinese Journal of Catalysis.

[7] Gong Y., Li H., Li C., Yang X., Wang J., Hosono H. (2022). LaRuSi Electride
Disrupts the Scaling Relations for Ammonia Synthesis. Chem. Mater..

[8] Kobayashi Y., Tada S., Mizoguchi H. (2021). Chemical Route
to Prepare Nickel
Supported on Intermetallic Ti_6_Si_7_Ni_16_ Nanoparticles Catalyzing CO Methanation. Nanoscale.

[9] Mizoguchi H., Park S.-W., Kishida K., Kitano M., Kim J., Sasase M., Honda T., Ikeda K., Otomo T., Hosono H. (2019). Zeolitic Intermetallics:
LnNiSi (Ln = La–Nd). J. Am. Chem. Soc..

[10] Wu J., Li J., Gong Y., Kitano M., Inoshita T., Hosono H. (2019). Intermetallic
Electride Catalyst as a Platform for Ammonia Synthesis. Angew. Chem., Int. Ed..

[11] Furukawa S., Komatsu T. (2017). Intermetallic Compounds:
Promising Inorganic Materials
for Well-Structured and Electronically Modified Reaction Environments
for Efficient Catalysis. ACS Catal..

[12] Brown, W. K. ; Plata, M. A. ; Raines, M. E. ; Chan, J. Y. Structural and Physical Properties of *R* _2_ *M* _3_ *X* _5_ Compounds. In Bunzli, J.-C. G. ; Kauzlarich, S. M. , Eds.; Handbook on the Physics and Chemistry of Rare Earths: Including Actinides; Elsevier, 2023; 64, 1–92.

[13] Villars, P. ; Cenzual, K. Pearson’s Crystal Data: Crystal Structure Database for Inorganic Compounds, Release 2024/25; ASM International: Materials Park, Ohio, USA, 2024.

[14] Braun H. F. (1980). Superconductivity
of Rare Earth-Iron Silicides. Phys. Lett. A.

[15] Singh Y., Ramakrishnan S. (2004). Magnetic Ordering and Superconductivity in the *R*
_2_Ir_3_Ge_5_(*R* = Y, La, Ce–Nd, Gd–Tm, Lu) System. Phys. Rev. B.

[16] Abliz M., Hedo M., Kitagawa J., Uwatoko Y., Ishikawa M. (2006). Pressure Induced
Kondo Coherence Effect. J. Alloys Compd..

[17] Hossain Z., Ohmoto H., Umeo K., Iga F., Suzuki T., Takabatake T., Takamoto N., Kindo K. (1999). Antiferromagnetic Kondo-Lattice
Systems Ce_2_Rh_3_Ge_5_ and Ce_2_Ir_3_Ge_5_ with Moderate Heavy-Fermion Behavior. Phys. Rev. B.

[18] Mazumdar C., Nigam A. K., Nagarajan R., Godart C., Gupta L. C., Padalia B. D., Chandra G., Vijayaraghavan R. (1996). Positive Giant
Magnetoresistance in Antiferromagnetic *RE*
_2_Ni_3_Si_5_ (*RE*=Tb, Sm, Nd). Appl. Phys. Lett..

[19] Ramakrishnan S., Schönleber A., Rekis T., van Well N., Noohinejad L., van Smaalen S., Tolkiehn M., Paulmann C., Bag B., Thamizhavel A., Pal D., Ramakrishnan S. (2020). Unusual Charge
Density Wave Transition and Absence of Magnetic Ordering in Er_2_Ir_3_Si_5_. Phys.
Rev. B.

[20] Brown W. K., Schundelmier B. C., Tan H., Melnick C., Kotliar G., Yan B., Wei K., McCandless G. T., Chan J. Y. (2025). Sm_2_Ru_3_Sn_5_: A Noncentrosymmetric
Cubic Member of the *Ln*
_2_
*M*
_3_
*X*
_5_ Family. Zeitschrift für
anorganische und allgemeine Chemie.

[21] Freccero R., De Negri S., Rogl G., Binder G., Michor H., Rogl P. F., Saccone A., Solokha P. (2021). La_2_Pd_3_Ge_5_ and Nd_2_Pd_3_Ge_5_ Compounds: Chemical Bonding and
Physical Properties. Inorg. Chem..

[22] Freccero R., Choi S. H., Solokha P., De Negri S., Takeuchi T., Hirai S., Mele P., Saccone A. (2019). Synthesis, Crystal
Structure and Physical Properties of Yb_2_Pd_3_Ge_5_. J. Alloys Compd..

[23] Solokha P., Freccero R., De Negri S., Proserpio D. M., Saccone A. (2016). The *R*
_2_Pd_3_Ge_5_ (*R* = La–Nd,
Sm) Germanides: Synthesis,
Crystal Structure and Symmetry Reduction. Struct
Chem..

[24] Freccero R., Solokha P., De Negri S. (2024). The La_2_Pd_3_(Si,
Ge)_5_ Complete Solid Solution: Crystal Structure, Chemical
Bonding, and Volume Chemistry. J. Alloys Compd..

[25] Dronskowski R., Blöchl P. E. (1993). Crystal
Orbital Hamilton Populations (COHP): Energy-Resolved
Visualization of Chemical Bonding in Solids Based on Density-Functional
Calculations. J. Phys. Chem..

[26] Gorelenko Y. K., Skolozdra R. V., Dutchak Y. I., Yarovets V. I., Shcherba I. D., Bodak O. I. (1985). Crystal
Structure X-Ray Spectra, Magnetic and Electrical
Properties of *R*
_2_Co_3_Si_5_ (R = Y, Gd, Tb, Dy, Ho, Er, Tm, Lu) Compounds. Ukrainskij Fizicheskij Zhurnal.

[27] Chabot B., Parthé E. (1985). Dy_2_Co_3_Si_5_, Lu_2_Co_3_Si_5_, Y_2_Co_3_Si_5_ and Sc_2_Co_3_Si_5_ with a Monoclinic
Structural Deformation Variant of the Orthorhombic U_2_Co_3_Si_5_ Structure Type. Journal
of the Less Common Metals.

[28] Kyrk T. M., Kennedy E. R., Galeano-Cabral J., McCandless G. T., Scott M. C., Baumbach R. E., Chan J. Y. (2024). Much more
to explore
with an oxidation state of nearly four: Pr valence instability in
intermetallic *m*-Pr_2_Co_3_Ge_5_. Sci. Adv..

[29] Méot-Meyer M., Venturini G., Malaman B., Mc Rae E., Roques B. (1985). Magnetisme
et Conductivite Des Siliciures Y_2_Mn_3_Si_5_ et Lu_2_Mn_3_Si_5_. Mater. Res. Bull..

[30] Segre, C. U. Superconductivity and Magnetism in Compounds with the Sc_2_Ru_3_Si_5_-Type Structure; Univ. of California: San Diego, CA, 1980. https://www.osti.gov/biblio/6314673.

[31] Bodak O. I., Pecharskii V. K., Gladyshevskii E. I. (1978). Y-Re-Si system and crystal structure
of certain new ternary compounds of rare earth metals. Izv. Akad. Nauk SSSR, Neorg. Mater. (USSR).

[32] Paccard D., Paccard L. (1987). Y_2_Rh_3_Si_5_, Dy_2_Rh_3_Si_5_ with a Monoclinic Structural Deformation
Variant of the Orthorhombic U_2_Co_3_Si_5_-Type Structure. Journal of the Less Common
Metals.

[33] Hirjak M., Lejay P., Chevalier B., Etourneau J., Hagenmuller P. (1985). Influence of Composition on the Structural and Superconducting
Properties of the Two Polymorphic Forms of Iridium- or Silicon-Substituted
YIr_2_Si_2_. Journal of the
Less Common Metals.

[34] Chabot B., Parthé E. (1984). Ce_2_Co_3_Si_5_ and *R*
_2_Ni_3_Si_5_ (*R* ≡ Ce, Dy, Y) with the Orthorhombic U_2_Co_3_Si_5_-Type Structure and the Structural
Relationship with
the tetragonaL Sc_2_Fe_3_Si_5_-Type Structure. Journal of the Less Common Metals.

[35] Patil S., Nagarajan R., Gupta L. C., Godart C., Vijayaraghavan R., Padalia B. D. (1988). ^151^Eu-Mössbauer Investigation in
Some New Ternary Eu_2_M_3_Si_5_ Systems
(*M* = Ni, Pd, Cu, Rh). Hyperfine
Interact..

[36] Kresse G., Hafner J. (1993). Ab Initio Molecular Dynamics for Liquid Metals. Phys. Rev. B.

[37] Kresse G., Furthmüller J. (1996). Efficiency of Ab-Initio Total Energy Calculations for
Metals and Semiconductors Using a Plane-Wave Basis Set. Comput. Mater. Sci..

[38] Kresse G., Furthmüller J. (1996). Efficient
Iterative Schemes for Ab Initio Total-Energy
Calculations Using a Plane-Wave Basis Set. Phys.
Rev. B.

[39] Kresse G., Joubert D. (1999). From Ultrasoft Pseudopotentials to
the Projector Augmented-Wave
Method. Phys. Rev. B.

[40] Hafner, J. ; Kresse, G. The Vienna AB-Initio Simulation Program VASP: An Efficient and Versatile Tool for Studying the Structural, Dynamic, and Electronic Properties of Materials. Properties of Complex Inorganic Solids; Springer: Boston, MA, 1997.

[41] Blöchl P. E. (1994). Projector
Augmented-Wave Method. Phys. Rev. B.

[42] Perdew J. P., Burke K., Ernzerhof M. (1996). Generalized Gradient Approximation
Made Simple. Phys. Rev. Lett..

[43] Monkhorst H. J., Pack J. D. (1976). Special Points for Brillouin-Zone Integrations. Phys. Rev. B.

[44] Pack J. D., Monkhorst H. J. (1977). “Special
Points for Brillouin-Zone Integrations”–a
Reply. Phys. Rev. B.

[45] Chevalier B., Lejay P., Etourneau J., Vlasse M., Hagenmuller P. (1982). Structure,
Superconductivity and Magnetism of New Rare Earth-Rhodium Silicides *RE*
_2_Rh_3_Si_5_ of U_2_Co_3_Si_5_-Type. Mater. Res.
Bull..

[46] Samsel-Czekała M., Winiarski M. J. (2012). Electronic Structure of Superconducting
Lu_2_Ni_3_Si_5_ and Its Reference Compound
Y_2_Ni_3_Si_5_ by Ab Initio Calculations. Intermetallics.

[47] Deringer V. L., Tchougréeff A. L., Dronskowski R. (2011). Crystal Orbital
Hamilton Population (COHP) Analysis As Projected from Plane-Wave Basis
Sets. J. Phys. Chem. A.

[48] Maintz S., Deringer V. L., Tchougréeff A.
L., Dronskowski R. (2016). LOBSTER: A
Tool to Extract Chemical Bonding from Plane-Wave Based DFT. J. Comput. Chem..

[49] Maintz S., Deringer V. L., Tchougréeff A.
L., Dronskowski R. (2013). Analytic Projection
from Plane-Wave and PAW Wavefunctions and Application to Chemical-Bonding
Analysis in Solids. J. Comput. Chem..

[50] Nelson R., Ertural C., George J., Deringer V., Hautier G., Dronskowski R. (2020). LOBSTER: Local
Orbital Projections, Atomic Charges,
and Chemical-bonding Analysis from Projector-augmented-wave-based
Density-functional Theory. J. Comput. Chem..

[51] Tang W., Sanville E., Henkelman G. (2009). A Grid-Based
Bader Analysis Algorithm
without Lattice Bias. J. Phys.: Condens. Matter.

[52] Henkelman G., Arnaldsson A., Jónsson H. (2006). A Fast and Robust Algorithm for Bader
Decomposition of Charge Density. Comput. Mater.
Sci..

[53] Sanville E., Kenny S. D., Smith R., Henkelman G. (2007). Improved Grid-Based
Algorithm for Bader Charge Allocation. J. Comput.
Chem..

[54] Yu M., Trinkle D. R. (2011). Accurate and Efficient Algorithm for Bader Charge Integration. J. Chem. Phys..

[55] Müller P. C., Ertural C., Hempelmann J., Dronskowski R. (2021). Crystal Orbital
Bond Index: Covalent Bond Orders in Solids. J. Phys. Chem. C.

[56] Gladisch F. C., Steinberg S. (2018). Revealing
Tendencies in the Electronic Structures of
Polar Intermetallic Compounds. Crystals.

[57] Steinberg S., Dronskowski R. (2018). The Crystal
Orbital Hamilton Population (COHP) Method
as a Tool to Visualize and Analyze Chemical Bonding in Intermetallic
Compounds. Crystals.

[58] Lin Q., Miller G. J. (2018). Electron-Poor Polar
Intermetallics: Complex Structures,
Novel Clusters, and Intriguing Bonding with Pronounced Electron Delocalization. Acc. Chem. Res..

[59] Smetana V., Steinberg S., Mudryk Y., Pecharsky V., Miller G. J., Mudring A.-V. (2015). Cation-Poor
Complex Metallic Alloys
in Ba­(Eu)–Au–Al­(Ga) Systems: Identifying the Keys That
Control Structural Arrangements and Atom Distributions at the Atomic
Level. Inorg. Chem..

[60] Smetana V., Lin Q., Pratt D. K., Kreyssig A., Ramazanoglu M., Corbett J. D., Goldman A. I., Miller G. J. (2012). A Sodium-Containing
Quasicrystal: Using Gold To Enhance Sodium’s Covalency in Intermetallic
Compounds. Angew. Chem., Int. Ed..

[61] Eck, B. wxDragon, 2020.

[62] Momma K., Izumi F. (2011). VESTA 3 for Three-Dimensional Visualization of Crystal, Volumetric
and Morphology Data. J. Appl. Crystallogr..

[63] Freccero R., Hübner J.-M., Prots Y., Schnelle W., Schmidt M., Wagner F. R., Schwarz U., Grin Y. (2021). “Excess”
Electrons in LuGe. Angew. Chem., Int. Ed..

[64] Hübner J.-M., Freccero R., Prots Y., Schwarz U. (2025). The Missing Link in
the Monogermanide Series: YbGe. *Chemistry–A*. European Journal.

[65] Martinelli A., Ryan D., Sereni J., Ritter C., Leineweber A., Čurlík I., Freccero R., Giovannini M. (2023). Magnetic Phase
Separation in the EuPdSn_2_ Ground State. J. Mater. Chem. C.

[66] Reitz L. S., Hempelmann J., Müller P. C., Dronskowski R., Steinberg S. (2024). Bonding Analyses
in the Broad Realm of Intermetallics:
Understanding the Role of Chemical Bonding in the Design of Novel
Materials. Chem. Mater..

[67] Weinelt L., Steinberg S. (2025). Exploring
the Correlation between Chemical Bonding
and Structural Distortions in TbCu_0.33_Te_2_. J. Phys.: Condens. Matter.

[68] Steinberg S. (2023). Revisiting
the Frontier of the Zintl–Klemm Approach for the Examples of
Three Mo_2_FeB_2_-Type Intermetallics by Means of
Quantumchemical Techniques. Zeitschrift für
anorganische und allgemeine Chemie.

[69] Repetto, G. Synthesis, flux growth and structural characterization of intermetallic silicides *R* _2_Pd_3_Si_5_ (*R* = rare earth metal). Master thesis, 2025. https://unire.unige.it/handle/123456789/11733.

