# Supplementary material for: Chemical Bonding Trends in Y2 M 3Si5 (M = Mn–Cu, Tc–Pd, Re–Pt): A Study within the Broad R 2 M 3 X 5 Intermetallic Family
Source: Inorg Chem. 2025 Oct 13;64(42):21067–75. doi: 10.1021/acs.inorgchem.5c03309 (PMC12570129; doi:10.1021/acs.inorgchem.5c03309)
Supplement: Supplementary file 1 [file ic5c03309_si_001.pdf]

Supporting Information

Chemical Bonding Trends in $Y_2M_3Si_5$ (M = Mn–Cu, Tc–Pd, Re–Pt): A Study within the Broad $R_2M_3X_5$ Intermetallic Family

Giorgio Palla[1], Linda S. Reitz,[2] Riccardo Freccero*[1], Serena De Negri[1], Richard Dronskowski[2]

1. Dipartimento di Chimica e Chimica Industriale, Università degli Studi di Genova, via Dodecaneso 31, 16146 Genova, Italy
2. Institute of Inorganic Chemistry, RWTH Aachen University, Landoltweg 1a, 52056 Aachen, Germany

* riccardo.freccero@unige.it

Crystal chemistry of the three considered structural prototypes

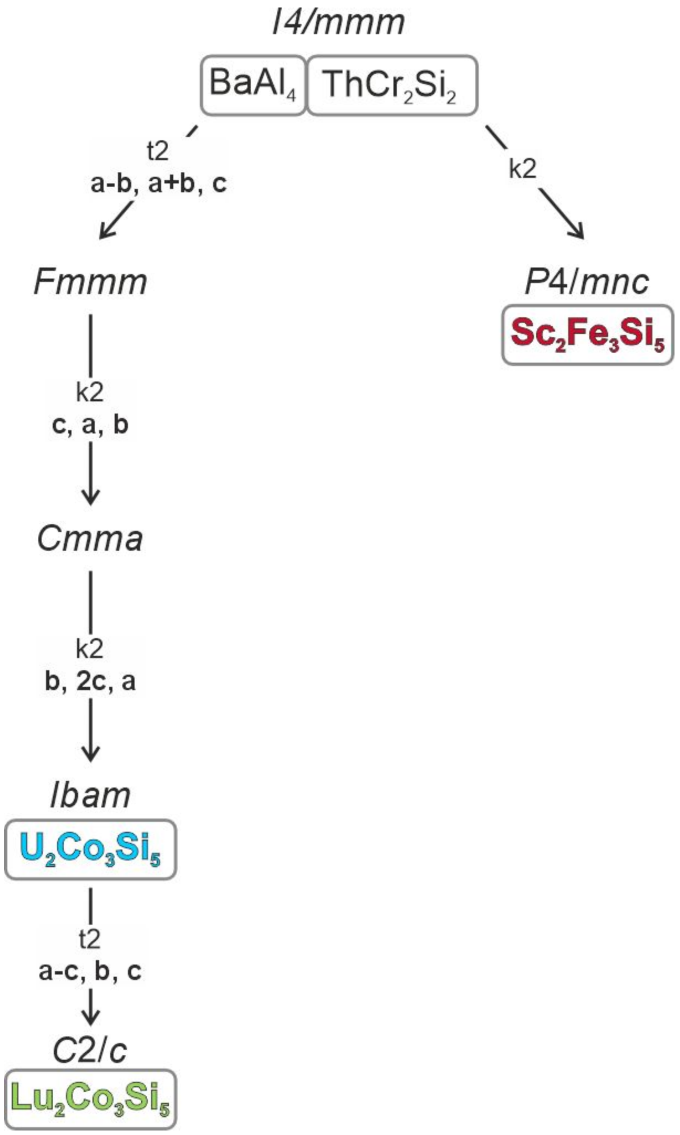

Figure S1. Bärninghausen tree relating the $BaAl_4$/$ThCr_2Si_2$ aristotype to the three most reported prototypes for $R_2M_3Si_5$ silicides.

The *tP*40-$Sc_2Fe_3Si_5$ structure type. This primitive tetragonal structure belongs to the *P*4/*mnc* space group, № 128 of the International Table of Crystallography. It consists of six symmetrically inequivalent atomic species occupying the 8*h*, 8*g*, 4*d* and 4*e* Wyckoff positions (Table S1).

Table S1. The experimentally determined atomic position for the $Y_2Fe_3Si_5$ [1] intermetallic compound are exemplarily reported.

Element	*Wick.*	*x*/*a*	*y*/*b*	*z*/*c*
Y	*8h*	0.260(6)	0.429(6)	0
Fe1	*8h*	0.159(9)	0.122(1)	0
Fe2	*4d*	0	½	¼
(2*b*)Si1	*8g*	0.183(9)	0.683(9)	¼
(1*b*)Si2	*4e*	0	0	0.257(0)
(2*b*)Si3	*8h*	0.014(8)	0.309(1)	0

Lattice parameters (Å)		
a	***b***	***c***
10.43	10.43	5.47

The *oI*40-$U_2Co_3Si_5$ structure type. This body centred orthorhombic structure belongs to the *Ibam* space group, № 72. Analogously to the *tP*40-$Sc_2Fe_3Si_5$ prototype it consists of six symmetrically inequivalent atomic species, occupying the 8*j*, 8*g*, 4*b* and 4*a* Wyckoff positions (Table S2).

Table S2. The experimentally determined atomic position for the $Y_2Ni_3Si_5$ [2] intermetallic compound are exemplarily reported.

Element	*Wick.*	*x*/*a*	*y*/*b*	*z*/*c*
Y	*8j*	0.263(2)	0.369(1)	0
Ni1	*8j*	0.112(3)	0.134(2)	0
Ni2	*4b*	½	0	¼
(2*b*)Si1	*8g*	0	0.266(3)	¼
(0*b*)Si2	*4a*	0	0	¼
(2*b*)Si3	*8j*	0.347(5)	0.107(4)	0

Lattice parameters (Å)		
a	***b***	***c***
6.01	9.60	11.12

The *mS*40–$Lu_2Co_3Si_5$ structure type. This one side centred monoclinic structure belongs to the *C*2/*c* space group, № 15. It is directly obtained through one symmetry reduction step from the *oI*40-$U_2Co_3Si_5$ type, as shown in the Bärninghausen tree (Figure S1). Thus, it consists of seven symmetrically inequivalent atomic species, occupying the 8*f* and 4*e* Wyckoff sites (Table S3), with two 4*e* Si position (i.e. Si1 and Si4) originating from the splitting of one Si1 8*g* position in the *oI*40 structure (Table S2), due to symmetry reduction[3].

Table S3. The experimentally determined atomic position for the $Y_2Co_3Si_5$ [4] intermetallic compound are exemplarily reported.

Element	*Wick.*	*x/a*	*y/b*	*z/c*
Y	*8f*	0.267(3)	0.137(5)	0.264(9)
Co1	*8f*	0.098(0)	0.359(2)	0.125(2)
Co2	*4e*	0	0.003(1)	¼
(2*b*)Si1	*4e*	0	0.213(4)	¼
(0*b*)Si2	*4e*	0	0.512(7)	¼
(2*b*)Si3	*8f*	0.332(3)	0.402(0)	0.330(0)
(2*b*)Si4	*4e*	0	0.773(7)	¼

Lattice parameters (Å)			
a	***a***	***c***	***β***
5.96	10.78	11.43	118.76°

1. Umarji A.M.; Malik S.K.; Shenoy G. Upper critical fields in $RE_2Fe_3Si_5$ (RE= Y, Sc, Lu) compounds. *J. Appl. Phys.,* **1985,** 57, 3118-3120.
2. Chabot B.; Parthe E. $Ce_2Co_3Si_5$ and $R_2Ni_3Si_5$ (R= Ce, Dy, Y) with the orthorhombic $U_2Co_3Si_5$-type structure and the structural relationship with the tetragonal $Sc_2Fe_3Si_5$-type structure. *J. Less-Common Met.*, **1984**, 97, 285-290.
3. Solokha, P.; Freccero, R.; De Negri, S.; Proserpio, D. M.; Saccone, A. The $R_2Pd_3Ge_5$ (R = La–Nd, Sm) Germanides: Synthesis, Crystal Structure and Symmetry Reduction. *Structural Chemistry* **2016**, *27* (6), 1693–1701.
4. Chabot, B.; Parthé, E. $Dy_2Co_3Si_5$, $Lu_2Co_3Si_5$, $Y_2Co_3Si_5$ and $Sc_2Co_3Si_5$ with a Monoclinic Structural Deformation Variant of the Orthorhombic $U_2Co_3Si_5$ Structure Type. *Journal of the Less Common Metals* **1985**, *106* (1), 53–59

Bonding data for all the calculated compounds

Table S4. All the calculated data. They are ordered per compound and structure. From left to right the columns correspond: 1) INTERACTION, 2) BOND LENGHT(Å), 3) –ICOHP (eV/bond), 4) ICOBI, 5) NUMBER OF INTERACTIONS IN THE CELL, 6) cum –ICOHP (eV/cell)

1)	2)	3)	4)	5)	6)
$Y_2\mathbf{Mn}_3Si_5$ *tP*40					
Mn1-Si3	2.341	1.73	0.38	8	13.88
Mn2-Si3	2.357	1.82	0.38	16	29.20
Mn1-Si1	2.364	1.77	0.39	16	28.43
Mn1-Si2	2.365	1.81	0.37	16	29.11
Mn1-Si3	2.394	1.72	0.38	8	13.82
Si1-Si3	2.480	2.53	0.36	16	40.50
Si2-Si2	2.570	2.15	0.27	2	4.31
Mn2-Si1	2.593	1.14	0.29	8	9.13
Mn2-Mn2	2.679	0.65	0.32	4	2.61
Si1-Si1	2.679	1.79	0.28	8	14.35
Y-Si3	2.750	2.49	0.37	8	19.99
Si2-Si2	2.788	1.82	0.30	2	3.66
Mn1-Mn1	2.808	0.43	0.20	8	3.49
Y-Si3	2.853	1.83	0.26	16	29.33
Y-Si2	2.942	1.66	0.26	16	26.70
Y-Si1	3.004	1.47	0.24	16	23.52
Y-Mn1	3.029	0.85	0.28	16	13.75
Y-Si1	3.075	1.13	0.18	16	18.22
Y-Mn2	3.157	0.53	0.17	16	8.56
Y-Mn1	3.402	0.33	0.15	8	2.70
Y-Mn1	3.504	0.26	0.10	16	4.27
Si2-Si3	3.551	0.16	0.03	16	2.62
Y-Y	3.664	0.50	0.09	8	4.05
$Y_2\mathbf{Mn}_3Si_5$ *oI*40					
Mn1-Si1	2.311	2.03	0.43	16	32.44
Mn1-Si3	2.313	2.20	0.52	8	17.61
Mn1-Si2	2.336	2.03	0.41	16	32.44
Mn2-Si3	2.401	1.73	0.36	16	27.64
Mn2-Si1	2.461	1.46	0.33	8	11.71
Si1-Si3	2.535	2.41	0.35	16	38.56
Mn2-Mn2	2.673	0.66	0.34	4	2.66
Si1-Si1	2.673	1.81	0.27	8	14.47
Si2-Si2	2.673	1.97	0.28	4	7.89
Y-Si3	2.849	1.91	0.29	16	30.54
Y-Si3	2.859	2.09	0.32	8	16.69
Y-Si1	2.937	1.46	0.22	16	23.39
Mn1-Y	2.941	0.89	0.24	8	7.14
Mn1-Y	3.019	0.85	0.26	16	13.55
Y-Si2	3.031	1.30	0.20	16	20.82
Mn1-Y	3.063	0.77	0.25	8	6.18
Mn1-Mn1	3.085	0.44	0.15	8	3.49
Y-Si1	3.138	1.05	0.17	16	16.72
Y-Si3	3.236	0.87	0.16	8	6.95
Mn2-Y	3.264	0.46	0.16	16	7.36
Si1-Si2	3.475	0.22	0.04	8	1.74
Mn1-Y	3.674	0.15	0.07	8	1.23
Y-Y	3.778	0.38	0.08	8	3.08
$Y_2\mathbf{Tc}_3Si_5$ *tP*40					
Tc1-Si3	2.445	2.14	0.41	8	17.16
Tc2-Si3	2.452	2.27	0.41	16	36.38
Tc1-Si1	2.458	2.31	0.44	16	36.95
Tc1-Si3	2.460	2.30	0.44	8	18.37
Tc1-Si2	2.503	2.15	0.39	16	34.42
Si1-Si3	2.563	2.25	0.33	16	35.97
Tc2-Si1	2.647	1.55	0.32	8	12.37
Si2-Si2	2.671	2.45	0.40	2	4.89
Tc2-Tc2	2.732	1.17	0.34	4	4.67
Si1-Si1	2.732	1.70	0.27	8	13.56
Si2-Si2	2.794	1.35	0.17	2	2.70
Y-Si3	2.826	2.22	0.34	8	17.76
Y-Si3	2.914	1.70	0.25	16	27.18
Tc1-Tc1	2.938	0.65	0.19	8	5.20
Y-Si2	3.019	1.51	0.25	16	24.19
Y-Tc1	3.092	1.11	0.31	16	17.83
Y-Si1	3.122	1.23	0.21	16	19.66
Y-Si1	3.199	0.92	0.16	16	14.76
Y-Tc2	3.283	0.61	0.16	16	9.74
Y-Tc1	3.496	0.41	0.14	8	3.31
Y-Tc1	3.629	0.31	0.10	16	4.96
Si2-Si3	3.662	0.15	0.03	16	2.43
Y-Y	3.829	0.38	0.08	8	3.06
$Y_2\mathbf{Tc}_3Si_5$ *oI*40					
Tc1-Si3	2.367	2.99	0.61	8	23.95
Tc1-Si1	2.411	2.56	0.48	16	40.95
Tc1-Si2	2.437	2.43	0.46	16	38.93
Tc2-Si3	2.507	2.11	0.39	16	33.83
Tc2-Si1	2.528	1.93	0.37	8	15.40
Si1-Si3	2.577	2.34	0.35	16	37.41
Tc2-Tc2	2.764	1.09	0.34	4	4.36
Si1-Si1	2.764	1.55	0.24	8	12.38
Si2-Si2	2.764	1.71	0.25	4	6.82
Y-Si3	2.915	1.76	0.27	16	28.16
Y-Si3	2.967	1.74	0.27	8	13.93
Y-Si1	2.999	1.36	0.21	16	21.71
Tc1-Y	3.074	1.03	0.24	8	8.20
Y-Si2	3.137	1.10	0.18	16	17.67
Tc1-Y	3.138	0.98	0.26	16	15.75
Tc1-Y	3.178	0.92	0.26	8	7.38
Tc1-Tc1	3.190	0.38	0.15	8	3.07
Tc2-Y	3.319	0.62	0.17	16	9.89
Y-Si1	3.319	0.76	0.13	16	12.19
Y-Si3	3.366	0.69	0.14	8	5.55
Si1-Si2	3.650	0.17	0.04	8	1.36
Tc1-Y	3.80	0.18	0.07	8	1.42
Y-Y	3.977	0.26	0.06	8	2.05
$Y_2\mathbf{Re}_3Si_5$ *tP*40					
Re1-Si3	2.456	2.73	0.49	8	21.82
Re1-Si3	2.461	2.35	0.42	8	18.81
Re1-Si1	2.467	2.58	0.47	16	41.33
Re2-Si3	2.470	2.46	0.42	16	39.42
Re1-Si2	2.526	2.37	0.39	16	37.85
Si1-Si3	2.590	2.14	0.30	16	34.29
Si2-Si2	2.624	2.71	0.43	2	5.42
Re2-Si1	2.657	1.74	0.34	8	13.91
Re2-Re2	2.736	1.73	0.41	4	6.91
Si1-Si1	2.736	1.73	0.26	8	13.82
Y-Si3	2.818	2.36	0.34	8	18.89
Si2-Si2	2.849	1.15	0.15	2	2.29
Y-Si3	2.911	1.77	0.25	16	28.33
Re1-Re1	2.949	0.88	0.21	8	7.05
Y-Si2	3.023	1.55	0.24	16	24.81
Y-Re1	3.103	1.19	0.31	16	18.98
Y-Si1	3.139	1.24	0.21	16	19.90
Y-Si1	3.206	0.93	0.15	16	14.83
Y-Re2	3.291	0.69	0.16	16	11.06
Y-Re1	3.515	0.43	0.14	8	3.42
Y-Re1	3.636	0.33	0.10	16	5.33
Si2-Si3	3.659	0.15	0.02	16	2.44
Y-Y	3.851	0.36	0.07	8	2.90
$Y_2\mathbf{Re}_3Si_5$ *oI*40					
Re1-Si3	2.366	3.41	0.66	8	27.31

Re1-Si1	2.418	2.83	0.49	16	45.26
Re1-Si2	2.458	2.58	0.46	16	41.33
Re2-Si3	2.527	2.27	0.39	16	36.38
Re2-Si1	2.538	2.13	0.37	8	17.04
Si1-Si3	2.593	2.29	0.33	16	36.58
Re2-Re2	2.758	1.57	0.43	4	6.29
Si1-Si1	2.758	1.57	0.25	8	12.54
Si2-Si2	2.758	1.77	0.25	4	7.08
Y-Si3	2.905	1.84	0.27	16	29.51
Y-Si3	2.990	1.73	0.26	8	13.86
Y-Si1	2.995	1.73	0.21	16	27.72
Re1-Y	3.110	1.07	0.23	8	8.58
Re1-Y	3.142	1.09	0.27	16	17.51
Y-Si2	3.167	1.07	0.17	16	17.15
Re1-Y	3.172	1.04	0.27	8	8.32
Re1-Re1	3.193	0.51	0.17	8	4.11
Re2-Y	3.329	0.68	0.18	16	10.89
Y-Si1	3.346	0.74	0.13	16	11.90
Y-Si3	3.382	0.68	0.13	8	5.44
Si1-Si2	3.686	0.13	0.04	8	1.04
Re1-Y	3.856	0.17	0.06	8	1.35
Y-Y	3.974	0.27	0.06	8	2.16
Y$_2$**Fe**$_3$Si$_5$ *tP*40					
Fe2-Si3	2.308	1.74	0.36	16	27.91
Fe1-Si2	2.322	1.72	0.35	16	27.52
Fe1-Si3	2.335	1.58	0.35	8	12.61
Fe1-Si1	2.340	1.59	0.32	16	25.37
Fe1-Si3	2.378	1.63	0.36	8	13.01
Si1-Si3	2.463	2.62	0.38	16	41.94
Si2-Si2	2.520	2.30	0.29	2	4.60
Fe2-Si1	2.581	1.07	0.28	8	8.52
Fe2-Fe2	2.709	0.43	0.24	4	1.71
Si1-Si1	2.709	1.66	0.27	8	13.28
Y-Si3	2.730	2.59	0.39	8	20.74
Fe1-Fe1	2.759	0.35	0.17	8	2.84
Y-Si3	2.882	1.71	0.25	16	27.34
Si2-Si2	2.898	1.47	0.26	2	2.94
Y-Si2	2.914	1.74	0.27	16	27.81
Y-Si1	2.991	1.52	0.25	16	24.28
Y-Si1	3.010	1.33	0.22	16	21.20
Y-Fe1	3.048	0.73	0.23	16	11.67
Y-Fe2	3.113	0.53	0.17	16	8.55
Y-Fe1	3.390	0.30	0.12	8	2.43
Y-Fe1	3.481	0.25	0.10	16	4.02
Si2-Si3	3.551	0.18	0.03	16	2.91
Y-Y	3.575	0.60	0.10	8	4.77
Y$_2$**Fe**$_3$Si$_5$ *oI*40					
Fe1-Si3	2.295	1.99	0.45	8	15.88
Fe1-Si1	2.305	1.77	0.36	16	28.28
Fe1-Si2	2.307	1.74	0.38	16	27.85
Fe2-Si3	2.342	1.67	0.35	16	26.75
Fe2-Si1	2.448	1.36	0.32	8	10.87
Si1-Si3	2.490	2.65	0.39	16	42.45
Fe2-Fe2	2.669	0.45	0.28	4	1.81
Si1-Si1	2.669	2.02	0.29	8	16.13
Si2-Si2	2.669	1.82	0.28	4	7.26
Y-Si3	2.841	2.14	0.33	8	17.14
Y-Si3	2.870	1.79	0.27	16	28.64
Y-Si1	2.954	1.40	0.22	16	22.41
Fe1-Y	2.973	0.87	0.28	16	13.93
Fe1-Y	2.975	0.72	0.21	8	5.80
Y-Si2	2.995	1.39	0.22	16	22.18
Fe1-Y	3.019	0.75	0.23	8	5.99
Y-Si1	3.073	1.16	0.18	16	18.51
Fe1-Fe1	3.148	0.12	0.08	8	0.94
Y-Si3	3.173	0.97	0.18	8	7.74
Fe2-Y	3.262	0.41	0.15	16	6.63
Si1-Si2	3.371	0.28	0.06	8	2.26
Fe1-Y	3.550	0.19	0.08	8	1.52
Y-Y	3.736	0.42	0.08	8	3.35
Y$_2$**Ru**$_3$Si$_5$ *tP*40					
Ru2-Si3	2.386	2.24	0.39	16	35.86
Ru1-Si1	2.438	2.05	0.35	16	32.75
Ru1-Si3	2.441	2.15	0.40	8	17.23
Ru1-Si3	2.445	1.93	0.36	8	15.40
Ru1-Si2	2.456	2.04	0.35	16	32.63
Si1-Si3	2.534	2.39	0.35	16	38.25
Ru2-Si1	2.612	1.48	0.31	8	11.87
Si2-Si2	2.766	1.37	0.17	2	2.74
Ru2-Ru2	2.784	0.62	0.20	4	2.48
Si1-Si1	2.784	1.52	0.26	8	12.14
Y-Si3	2.794	2.33	0.35	8	18.63
Si2-Si2	2.801	1.95	0.36	2	3.89
Ru1-Ru1	2.871	0.50	0.15	8	4.02
Y-Si3	2.964	1.51	0.23	16	24.18
Y-Si2	2.973	1.63	0.26	16	26.08
Y-Si1	3.110	1.15	0.20	16	18.32
Y-Si1	3.120	1.25	0.22	16	20.05
Y-Ru1	3.127	0.90	0.23	16	14.34
Y-Ru2	3.228	0.60	0.15	16	9.63
Y-Ru1	3.480	0.38	0.12	8	3.01
Y-Ru1	3.607	0.27	0.08	16	4.39
Si2-Si3	3.632	0.19	0.04	16	3.10
Y-Y	3.709	0.48	0.09	8	3.86
Y$_2$**Ru**$_3$Si$_5$ *oI*40					
Ru1-Si3	2.356	2.66	0.50	8	21.28
Ru1-Si1	2.405	2.24	0.41	16	35.87
Ru1-Si2	2.409	2.21	0.38	16	35.32
Ru2-Si3	2.425	2.10	0.37	16	33.56
Ru2-Si1	2.500	1.82	0.35	8	14.55
Si1-Si3	2.524	2.66	0.40	16	42.63
Ru2-Ru2	2.777	0.61	0.21	4	2.44
Si1-Si1	2.777	1.72	0.26	8	13.79
Si2-Si2	2.777	1.53	0.25	4	6.14
Y-Si3	2.934	1.83	0.29	8	14.61
Y-Si3	2.969	1.54	0.24	16	24.71
Y-Si1	3.036	1.25	0.20	16	19.99
Y-Si2	3.086	1.21	0.19	16	19.29
Ru1-Y	3.088	1.00	0.27	16	16.03
Ru1-Y	3.107	0.80	0.19	8	6.37
Ru1-Y	3.119	0.90	0.22	8	7.17
Y-Si1	3.230	0.90	0.15	16	14.33
Ru1-Ru1	3.289	0.16	0.06	8	1.27
Y-Si3	3.291	0.81	0.16	8	6.49
Ru2-Y	3.333	0.52	0.15	16	8.40
Si1-Si2	3.516	0.26	0.06	8	2.06
Ru1-Y	3.641	0.23	0.08	8	1.87
Y-Y	3.928	0.28	0.07	8	2.28
Y$_2$**Os**$_3$Si$_5$ *tP*40					
Os2-Si3	2.400	2.40	0.42	16	38.37
Os1-Si3	2.432	2.46	0.45	8	19.66
Os1-Si1	2.444	2.29	0.40	16	36.68
Os1-Si3	2.464	2.07	0.38	8	16.58
Os1-Si2	2.487	2.18	0.37	16	34.83
Si1-Si3	2.555	2.18	0.31	16	34.88
Os2-Si1	2.607	1.73	0.35	8	13.81
Y-Si3	2.786	2.32	0.35	8	18.58
Si2-Si2	2.809	1.87	0.34	2	3.75
Os2-Os2	2.811	0.79	0.22	4	3.15
Si1-Si1	2.811	1.40	0.24	8	11.16
Si2-Si2	2.813	1.21	0.15	2	2.42
Os1-Os1	2.900	0.64	0.16	8	5.13
Y-Si2	2.978	1.59	0.25	16	25.43
Y-Si3	2.987	1.41	0.21	16	22.59
Y-Si1	3.114	1.14	0.20	16	18.25
Y-Si1	3.146	1.18	0.21	16	18.95
Y-Os1	3.151	0.97	0.25	16	15.59
Y-Os2	3.240	0.67	0.17	16	10.71
Y-Os1	3.479	0.41	0.12	8	3.28
Y-Os1	3.633	0.29	0.09	16	4.62
Si2-Si3	3.640	0.18	0.03	16	2.93
Y-Y	3.715	0.48	0.09	8	3.87

$Y_2\mathbf{Os}_3Si_5$ *oI*40					
Os1-Si3	2.346	2.98	0.57	8	21.28
Os1-Si1	2.421	2.41	0.41	16	35.87
Os1-Si2	2.433	2.36	0.43	16	35.32
Os2-Si3	2.434	2.30	0.40	16	33.56
Os2-Si1	2.507	2.04	0.39	8	14.55
Si1-Si3	2.533	2.56	0.38	16	42.63
Os2-Os2	2.806	0.78	0.23	4	2.44
Si1-Si1	2.806	1.61	0.25	8	13.79
Si2-Si2	2.806	1.40	0.23	4	6.14
Y-Si3	2.943	1.78	0.28	8	14.61
Y-Si3	2.999	1.42	0.22	16	24.71
Y-Si1	3.052	1.20	0.19	16	19.99
Y-Si2	3.100	1.16	0.18	16	19.29
Os1-Y	3.102	1.05	0.26	8	16.03
Os1-Y	3.103	1.11	0.30	16	6.37
Os1-Y	3.147	0.83	0.20	8	7.17
Y-Si1	3.240	0.87	0.14	16	14.33
Y-Si3	3.293	0.78	0.15	8	1.27
Os1-Os1	3.347	0.16	0.06	8	6.49
Os2-Y	3.348	0.58	0.16	16	8.40
Si1-Si2	3.516	0.22	0.05	8	2.06
Os1-Y	3.645	0.26	0.08	8	1.87
Y-Y	3.950	0.28	0.07	8	2.28

$Y_2\mathbf{Os}_3Si_5$ *mS*40					
Os2-Si3	2.331	3.09	0.58	8	24.75
Os2-Si4	2.355	2.74	0.47	4	10.95
Os2-Si3	2.402	2.47	0.43	8	19.78
Os1-Si2	2.417	2.40	0.44	8	19.19
Os1-Si4	2.418	2.52	0.46	8	20.13
Os2-Si1	2.457	2.27	0.40	4	9.07
Os1-Si2	2.458	2.27	0.41	8	18.14
Os1-Si3	2.462	2.21	0.38	8	17.66
Si4-Si3	2.533	2.49	0.35	8	19.90
Si1-Si3	2.583	2.48	0.38	8	19.80
Os1-Si1	2.678	1.48	0.29	8	11.86
Os1-Os1	2.868	0.73	0.22	4	2.91
Si1-Si4	2.877	1.18	0.20	8	9.41
Si2-Si2	2.885	1.28	0.19	4	5.13
Y-Si3	2.896	1.95	0.30	8	15.64
Y-Si2	2.962	1.54	0.24	8	12.35
Y-Si3	3.030	1.36	0.22	8	10.90
Y-Si4	3.034	1.27	0.19	8	10.14
Y-Si3	3.081	1.21	0.19	8	9.65
Os1-Y	3.082	1.10	0.26	8	8.79
Os1-Y	3.090	1.12	0.28	8	8.96
Y-Si1	3.090	1.20	0.19	8	9.59
Si1-Si2	3.096	0.64	0.09	4	2.54
Os2-Os2	3.101	0.39	0.12	4	1.58
Y-Si1	3.140	1.04	0.18	8	8.31
Os1-Y	3.213	0.86	0.23	8	6.84
Os1-Y	3.239	0.69	0.17	8	5.50
Y-Si3	3.250	0.86	0.16	8	6.88
Y-Si2	3.255	0.84	0.14	8	6.69
Os2-Y	3.302	0.63	0.16	8	5.06
Y-Si4	3.347	0.69	0.12	8	5.55
Os1-Y	3.351	0.59	0.16	8	4.68
Os2-Y	3.528	0.35	0.10	8	2.77
Y-Y	3.910	0.30	0.08	4	1.18

$Y_2\mathbf{Co}_3Si_5$ *tP*40					
Co2-Si3	2.302	1.59	0.31	16	25.38
Co1-Si1	2.341	1.36	0.26	16	21.71
Co1-Si2	2.346	1.51	0.29	16	24.19
Co1-Si3	2.352	1.37	0.29	8	10.98
Co1-Si3	2.369	1.50	0.31	8	11.97
Si1-Si3	2.465	2.71	0.40	16	43.30
Co2-Si1	2.607	0.85	0.20	8	6.78
Si2-Si2	2.624	2.02	0.26	2	4.03
Co2-Co2	2.698	0.30	0.13	4	1.20
Si1-Si1	2.698	1.75	0.29	8	14.03
Y-Si3	2.735	2.61	0.39	8	20.86
Co1-Co1	2.749	0.24	0.10	8	1.89
Si2-Si2	2.771	1.92	0.34	2	3.83
Y-Si3	2.870	1.79	0.27	16	28.69
Y-Si2	2.890	1.81	0.29	16	28.95
Y-Si1	2.981	1.57	0.27	16	25.12
Y-Si1	3.008	1.37	0.24	16	21.98
Y-Co1	3.036	0.64	0.18	16	10.31
Y-Co2	3.101	0.47	0.13	16	7.59
Y-Co1	3.393	0.25	0.09	8	2.03
Y-Co1	3.482	0.21	0.07	16	3.31
Si2-Si3	3.551	0.19	0.04	16	3.12
Y-Y	3.587	0.57	0.11	8	4.60

$Y_2\mathbf{Co}_3Si_5$ *oI*40					
Co1-Si3	2.243	1.92	0.39	16	30.77
Co1-Si1	2.285	1.62	0.31	16	25.97
Co1-Si2	2.296	1.60	0.32	16	25.56
Co2-Si3	2.320	1.57	0.30	8	12.56
Si1-Si3	2.473	2.85	0.43	16	45.57
Co2-Si1	2.515	1.02	0.24	4	4.10
Co2-Co2	2.735	0.33	0.16	8	2.63
Si1-Si1	2.735	1.55	0.26	16	24.74
Si2-Si2	2.735	1.83	0.28	16	29.22
Y-Si3	2.809	2.32	0.35	8	18.59
Y-Si3	2.940	1.58	0.25	8	12.62
Co1-Y	2.954	0.74	0.18	8	5.92
Co1-Y	2.985	0.73	0.20	16	11.64
Y-Si2	2.996	1.42	0.23	16	22.75
Y-Si1	3.027	1.26	0.20	8	10.07
Y-Si1	3.031	1.28	0.21	8	10.22
Y-Si3	3.053	1.25	0.22	8	9.98
Si1-Si2	3.083	0.69	0.11	16	11.11
Co1-Y	3.197	0.39	0.13	8	3.09
Co2-Y	3.235	0.39	0.12	16	6.20
Co1-Y	3.286	0.32	0.10	8	2.59
Co1-Co1	3.388	0.03	0.04	8	0.25
Y-Y	3.770	0.39	0.07	8	3.13

$Y_2\mathbf{Co}_3Si_5$ *mS*40					
Co1-Si4	2.247	1.75	0.33	8	13.98
Co1-Si3	2.262	1.85	0.37	8	14.82
Co1-Si2	2.268	1.71	0.35	8	13.67
Co1-Si2	2.299	1.57	0.32	8	12.53
Co2-Si3	2.312	1.61	0.31	8	12.91
Co1-Si1	2.329	1.47	0.29	8	11.80
Co2-Si3	2.349	1.47	0.29	8	11.79
Co2-Si4	2.398	1.25	0.27	4	4.99
Si4-Si3	2.465	2.78	0.41	8	22.28
Si1-Si3	2.491	2.83	0.45	8	22.68
Co2-Si1	2.622	0.82	0.20	4	3.28
Co2-Co2	2.726	0.33	0.16	4	1.33
Si1-Si4	2.730	1.56	0.25	8	12.46
Si2-Si2	2.736	1.77	0.27	4	7.08
Y-Si3	2.822	2.28	0.35	8	18.24
Y-Si2	2.905	1.68	0.26	8	13.48
Y-Si3	2.916	1.67	0.27	8	13.37
Co1-Y	2.921	0.80	0.21	8	6.43
Y-Si3	2.929	1.61	0.25	8	12.91
Si1-Si2	2.936	0.96	0.15	4	3.85
Y-Si4	2.950	1.51	0.24	8	12.11
Y-Si1	2.955	0.69	0.23	8	5.49
Co1-Y	2.986	0.69	0.17	8	5.50
Co1-Co1	3.050	0.09	0.05	4	0.35
Y-Si1	3.061	1.19	0.20	8	9.51
Y-Si3	3.065	1.23	0.22	8	9.80
Co1-Y	3.074	0.63	0.19	8	5.04
Y-Si2	3.094	1.17	0.19	8	9.33
Y-Si4	3.108	1.08	0.17	8	8.61
Co1-Y	3.131	0.45	0.14	8	3.62
Co2-Y	3.187	0.43	0.14	8	3.47
Co2-Y	3.274	0.35	0.11	8	2.82
Si2-Si4	3.330	0.37	0.07	4	1.48
Co1-Y	3.340	0.28	0.09	8	2.24

Y-Y	3.753	0.39	0.08	4	1.58
Y-Y	3.769	0.40	0.09	4	1.61
Y_2**Rh**$_3Si_5$ *tP*40					
Rh2-Si3	2.397	1.94	0.32	16	31.04
Rh1-Si3	2.410	2.02	0.35	8	16.14
Rh1-Si1	2.443	1.69	0.27	16	27.02
Rh1-Si3	2.465	1.62	0.28	8	12.95
Si2-Si2	2.476	3.43	0.59	2	6.86
Rh1-Si2	2.537	1.57	0.25	16	25.15
Si1-Si3	2.541	2.49	0.38	16	39.84
Rh2-Si1	2.650	1.13	0.23	8	9.05
Rh2-Rh2	2.764	0.47	0.13	4	1.88
Si1-Si1	2.764	1.67	0.30	8	13.38
Y-Si3	2.816	2.29	0.35	8	18.32
Rh1-Rh1	2.867	0.35	0.09	8	2.82
Y-Si2	2.912	1.76	0.30	16	28.22
Y-Si3	2.939	1.66	0.26	16	26.51
Si2-Si2	3.052	0.84	0.11	2	1.67
Y-Rh1	3.102	0.79	0.18	16	12.71
Y-Si1	3.110	1.31	0.24	16	20.89
Y-Si1	3.113	1.21	0.23	16	19.35
Y-Rh2	3.221	0.52	0.12	16	8.33
Y-Rh1	3.488	0.32	0.09	8	2.52
Y-Rh1	3.612	0.22	0.06	16	3.48
Si2-Si3	3.649	0.21	0.04	16	3.29
Y-Y	3.728	0.45	0.10	8	3.57
Y_2**Rh**$_3Si_5$ *oI*40					
Rh1-Si3	2.320	2.42	0.40	8	19.36
Rh1-Si1	2.391	1.97	0.32	16	31.50
Rh1-Si2	2.397	1.96	0.33	16	31.36
Rh2-Si3	2.426	1.84	0.30	16	29.49
Si1-Si3	2.499	2.93	0.46	16	46.89
Rh2-Si1	2.549	1.38	0.26	8	11.08
Rh2-Rh2	2.848	0.44	0.13	4	1.78
Si1-Si1	2.848	1.29	0.23	8	10.36
Si2-Si2	2.848	1.58	0.26	4	6.32
Y-Si3	2.933	1.93	0.32	8	15.47
Y-Si3	3.060	1.33	0.22	16	21.35
Rh1-Y	3.061	0.86	0.17	8	6.86
Y-Si2	3.081	1.29	0.22	16	20.70
Rh1-Y	3.096	0.82	0.18	16	13.09
Y-Si3	3.121	1.17	0.22	8	9.35
Y-Si1	3.155	1.07	0.19	16	17.05
Y-Si1	3.167	1.02	0.17	16	16.29
Si1-Si2	3.220	0.59	0.10	8	4.71
Rh1-Y	3.322	0.42	0.11	8	3.38
Rh2-Y	3.353	0.44	0.11	16	7.03
Rh1-Y	3.366	0.39	0.10	8	3.10
Rh1-Rh1	3.544	0.04	0.03	8	0.31
Y-Y	3.951	0.27	0.07	8	2.15
Y_2**Rh**$_3Si_5$ *mS*40					
Rh1-Si3	2.338	2.33	0.39	8	18.65
Rh1-Si4	2.352	2.14	0.34	8	17.11
Rh1-Si2	2.369	2.11	0.37	8	16.88
Rh1-Si2	2.394	1.94	0.33	8	15.55
Rh2-Si3	2.411	1.90	0.31	8	15.22
Rh2-Si4	2.445	1.66	0.29	4	6.64
Rh1-Si1	2.452	1.73	0.29	8	13.82
Rh2-Si3	2.467	1.69	0.28	8	13.54
Si4-Si3	2.495	2.84	0.43	8	22.74
Si1-Si3	2.510	2.93	0.48	8	23.47
Rh2-Si1	2.636	1.14	0.23	4	4.55
Rh2-Rh2	2.825	0.45	0.13	4	1.79
Si1-Si4	2.830	1.35	0.23	8	10.80
Si2-Si2	2.838	1.57	0.25	4	6.27
Y-Si3	2.957	1.86	0.31	8	14.85
Y-Si2	2.969	1.60	0.26	8	12.81
Y-Si3	3.014	1.48	0.25	8	11.80
Y-Si3	3.031	1.41	0.23	8	11.29
Y-Si4	3.051	1.32	0.22	8	10.53
Rh1-Y	3.053	0.88	0.19	8	7.06

Y-Si1	3.085	1.21	0.21	8	9.71
Si1-Si2	3.089	0.77	0.12	4	3.07
Rh1-Y	3.099	0.77	0.16	8	6.19
Rh1-Rh1	3.142	0.14	0.04	4	0.58
Y-Si3	3.144	1.13	0.22	8	9.04
Rh1-Y	3.155	0.74	0.18	8	5.94
Y-Si1	3.165	1.03	0.19	8	8.27
Y-Si2	3.221	0.96	0.17	8	7.69
Rh1-Y	3.249	0.50	0.13	8	4.03
Y-Si4	3.282	0.80	0.14	8	6.43
Rh2-Y	3.305	0.48	0.12	8	3.86
Rh2-Y	3.379	0.41	0.11	8	3.31
Rh1-Y	3.453	0.31	0.08	8	2.48
Si2-Si4	3.523	0.31	0.07	4	1.23
Y-Y	3.891	0.30	0.07	4	1.21
Y-Y	3.967	0.27	0.07	4	1.07
Y_2**Ir**$_3Si_5$ *tP*40					
Ir1-Si3	2.389	2.37	0.40	8	18.95
Ir2-Si3	2.406	2.14	0.35	16	34.19
Ir1-Si1	2.442	1.92	0.31	16	30.73
Si2-Si2	2.465	3.31	0.56	2	6.61
Ir1-Si3	2.487	1.76	0.30	8	14.09
Si1-Si3	2.566	2.28	0.33	16	36.44
Ir1-Si2	2.571	1.69	0.27	16	27.01
Ir2-Si1	2.644	1.36	0.27	8	10.84
Ir2-Ir2	2.789	0.61	0.17	4	2.43
Si1-Si1	2.789	1.57	0.28	8	12.56
Y-Si3	2.811	2.26	0.33	8	18.04
Y-Si2	2.894	1.78	0.30	16	28.53
Ir1-Ir1	2.894	0.46	0.11	8	3.65
Y-Si3	2.965	1.55	0.24	16	24.77
Si2-Si2	3.114	0.74	0.10	2	1.48
Y-Ir1	3.116	0.88	0.21	16	14.15
Y-Si1	3.119	1.21	0.23	16	19.41
Y-Si1	3.121	1.27	0.23	16	20.31
Y-Ir2	3.242	0.57	0.13	16	9.14
Y-Ir1	3.480	0.34	0.10	8	2.74
Y-Ir1	3.632	0.23	0.07	16	3.61
Si2-Si3	3.644	0.21	0.04	16	3.42
Y-Y	3.702	0.48	0.11	8	3.87
Y_2**Ir**$_3Si_5$ *oI*40					
Ir1-Si3	2.315	2.65	0.44	8	19.36
Ir1-Si1	2.391	2.22	0.37	16	31.50
Ir1-Si2	2.426	2.08	0.35	16	31.36
Ir2-Si3	2.435	2.06	0.33	16	29.49
Si1-Si3	2.520	2.78	0.42	16	46.89
Ir2-Si1	2.565	1.59	0.30	8	11.08
Ir2-Ir2	2.891	0.55	0.17	4	1.78
Si1-Si1	2.891	1.15	0.20	8	10.36
Si2-Si2	2.891	1.39	0.22	4	6.32
Y-Si3	2.920	1.98	0.32	8	15.47
Ir1-Y	3.038	1.04	0.22	8	21.35
Y-Si2	3.081	1.28	0.22	16	6.86
Y-Si3	3.086	1.24	0.22	8	20.70
Ir1-Y	3.113	0.90	0.20	16	13.09
Y-Si3	3.119	1.17	0.19	16	9.35
Si1-Si2	3.124	0.72	0.11	8	17.05
Y-Si1	3.152	1.04	0.18	16	16.29
Y-Si1	3.231	0.92	0.17	16	4.71
Ir1-Y	3.287	0.53	0.13	8	3.38
Ir2-Y	3.392	0.46	0.12	16	7.03
Ir1-Y	3.404	0.40	0.11	8	3.10
Ir1-Ir1	3.666	0.04	0.04	8	0.31
Y-Y	3.983	0.27	0.07	8	2.15
Y_2**Ni**$_3Si_5$ *tP*40					
Ni2-Si3	2.320	1.36	0.24	16	21.76
Ni1-Si1	2.373	1.09	0.19	16	17.42
Ni1-Si3	2.378	1.32	0.25	8	10.59
Ni1-Si3	2.406	1.06	0.20	8	8.46
Ni1-Si2	2.424	1.24	0.22	16	19.81
Si1-Si3	2.479	2.80	0.45	16	44.81

Si2-Si2	2.553	2.85	0.49	2	5.70
Ni2-Si1	2.668	0.59	0.13	8	4.72
Ni2-Ni2	2.702	0.18	0.06	4	0.71
Si1-Si1	2.702	1.82	0.30	8	14.57
Ni1-Ni1	2.773	0.16	0.06	8	1.25
Y-Si3	2.776	2.51	0.40	8	20.04
Si2-Si2	2.850	1.42	0.21	2	2.84
Y-Si3	2.870	1.89	0.30	16	30.31
Y-Si2	2.895	1.82	0.32	16	29.08
Y-Si1	3.016	1.52	0.27	16	24.39
Y-Si1	3.026	1.35	0.24	16	21.63
Y-Ni1	3.048	0.53	0.12	16	8.49
Y-Ni2	3.101	0.41	0.11	16	6.58
Y-Ni1	3.432	0.18	0.06	8	1.44
Y-Ni1	3.527	0.15	0.05	16	2.40
Si2-Si3	3.602	0.17	0.03	16	2.68
Y-Y	3.674	0.51	0.10	8	4.04
Y$_2$**Ni**$_3$Si$_5$ *oI*40					
Ni1-Si3	2.251	1.64	0.29	8	13.15
Ni1-Si1	2.285	1.42	0.25	16	22.69
Ni1-Si2	2.299	1.36	0.24	16	21.76
Ni2-Si3	2.334	1.37	0.24	16	21.92
Si1-Si3	2.456	3.01	0.48	16	48.22
Ni2-Si1	2.579	0.72	0.15	8	5.80
Ni2-Ni2	2.799	0.14	0.05	4	0.56
Si1-Si1	2.799	1.33	0.23	8	10.63
Si2-Si2	2.799	1.62	0.26	4	6.47
Y-Si3	2.827	2.33	0.37	8	18.63
Si1-Si2	2.932	1.12	0.18	8	8.94
Ni1-Y	2.955	0.62	0.13	16	9.91
Y-Si3	2.995	1.44	0.26	8	11.56
Y-Si3	3.002	1.45	0.24	16	23.26
Y-Si2	3.021	1.45	0.26	16	23.14
Y-Si1	3.037	1.25	0.21	8	10.02
Ni1-Y	3.038	0.54	0.12	16	8.61
Y-Si1	3.081	1.21	0.22	16	19.40
Ni1-Y	3.155	0.37	0.09	8	2.93
Ni2-Y	3.212	0.36	0.10	16	5.78
Ni1-Y	3.321	0.24	0.09	8	1.92
Y-Y	3.838	0.37	0.08	8	2.96
Y$_2$**Pd**$_3$Si$_5$ *tP*40					
Si2-Si2	2.377	4.10	0.72	2	8.21
Pd1-Si3	2.405	1.66	0.27	8	13.26
Pd2-Si3	2.429	1.44	0.22	16	22.98
Pd1-Si1	2.506	1.14	0.17	16	18.21
Si1-Si3	2.546	2.70	0.47	16	43.24
Pd1-Si3	2.551	1.03	0.17	8	8.22
Pd1-Si2	2.617	1.13	0.18	16	18.04
Pd2-Si1	2.725	0.67	0.12	8	5.36
Pd2-Pd2	2.803	0.18	0.04	4	0.73
Si1-Si1	2.803	1.60	0.30	8	12.80
Y-Si3	2.901	2.11	0.37	8	16.91
Pd1-Pd1	2.912	0.16	0.04	8	1.31
Y-Si2	2.960	1.69	0.32	16	27.01
Y-Si3	2.975	1.67	0.29	16	26.73
Y-Si1	3.133	1.22	0.25	16	19.55
Y-Pd1	3.137	0.55	0.11	16	8.83
Y-Si1	3.182	1.23	0.24	16	19.63
Si2-Si2	3.229	0.62	0.10	2	1.25
Y-Pd2	3.231	0.39	0.09	16	6.27
Y-Pd1	3.537	0.19	0.05	8	1.52
Y-Pd1	3.709	0.12	0.04	16	1.93
Si2-Si3	3.764	0.15	0.04	16	2.46
Y-Y	3.834	0.40	0.09	8	3.18
Y$_2$**Pd**$_3$Si$_5$ *oI*40					
Pd1-Si3	2.347	1.78	0.27	8	14.20
Pd1-Si1	2.409	1.51	0.24	16	24.23
Pd1-Si2	2.422	1.45	0.23	16	23.13
Si1-Si3	2.467	3.23	0.54	16	51.66
Pd2-Si3	2.472	1.34	0.21	16	21.43
Pd2-Si1	2.582	0.95	0.16	8	7.57

Pd2-Pd2	2.930	0.15	0.04	4	0.59
Si1-Si1	2.930	1.06	0.21	8	8.47
Si2-Si2	2.930	1.36	0.25	4	5.44
Y-Si3	2.988	1.86	0.34	8	14.92
Y-Si3	3.066	1.36	0.27	8	10.91
Pd1-Y	3.085	0.60	0.11	8	4.83
Y-Si2	3.101	1.37	0.27	16	22.00
Y-Si3	3.144	1.21	0.22	16	19.31
Si1-Si2	3.147	0.80	0.15	8	6.37
Pd1-Y	3.169	0.51	0.10	16	8.20
Y-Si1	3.203	0.97	0.18	16	15.46
Y-Si1	3.215	1.01	0.20	16	16.12
Pd1-Y	3.283	0.36	0.08	8	2.84
Pd2-Y	3.357	0.33	0.08	16	5.35
Pd1-Y	3.406	0.26	0.07	8	2.05
Pd1-Pd1	3.665	0.01	0.01	8	0.09
Y-Y	4.057	0.25	0.07	8	2.00
Y$_2$**Pt**$_3$Si$_5$ *tP*40					
Pt1-Si3	2.355	2.23	0.35	8	17.87
Pt2-Si3	2.430	1.78	0.26	16	28.48
Pt1-Si1	2.457	1.60	0.24	16	25.57
Si2-Si2	2.466	3.26	0.57	2	6.53
Si1-Si3	2.577	2.38	0.39	16	38.16
Pt1-Si3	2.604	1.13	0.18	8	9.02
Pt1-Si2	2.683	1.25	0.19	16	19.95
Pt2-Si1	2.739	0.81	0.14	8	6.50
Pt2-Pt2	2.824	0.26	0.05	4	1.02
Si1-Si1	2.824	1.53	0.28	8	12.24
Y-Si3	2.933	1.93	0.32	8	15.40
Y-Si2	2.944	1.72	0.33	16	27.53
Y-Si3	2.996	1.55	0.26	16	24.82
Pt1-Pt1	3.055	0.18	0.04	8	1.41
Y-Si1	3.111	1.29	0.26	16	20.63
Y-Pt1	3.137	0.73	0.15	16	11.75
Si2-Si2	3.182	0.79	0.13	2	1.59
Y-Si1	3.222	1.10	0.21	16	17.62
Y-Pt2	3.256	0.48	0.10	16	7.71
Y-Pt1	3.497	0.26	0.07	8	2.10
Y-Pt1	3.731	0.15	0.04	16	2.33
Si2-Si3	3.753	0.19	0.04	16	3.08
Y-Y	3.780	0.43	0.10	8	3.42
Y$_2$**Pt**$_3$Si$_5$ *oI*40					
Pt1-Si3	2.350	2.15	0.31	8	17.23
Pt1-Si1	2.411	1.86	0.29	16	29.72
Pt1-Si2	2.426	1.76	0.27	16	28.10
Pt2-Si3	2.471	1.67	0.25	16	26.73
Si1-Si3	2.489	3.01	0.49	16	48.14
Pt2-Si1	2.578	1.23	0.21	8	9.85
Y-Si3	2.974	1.90	0.34	8	15.18
Pt2-Pt2	2.988	0.16	0.04	4	0.66
Si1-Si1	2.988	0.94	0.18	8	7.50
Si2-Si2	2.988	1.19	0.21	4	4.75
Y-Si3	3.014	1.49	0.29	8	11.90
Pt1-Y	3.061	0.84	0.15	8	6.73
Si1-Si2	3.064	0.94	0.15	8	7.53
Y-Si2	3.095	1.33	0.25	16	21.24
Y-Si3	3.166	1.03	0.19	16	16.55
Pt1-Y	3.205	0.61	0.12	16	9.82
Y-Si1	3.216	1.02	0.19	16	16.31
Pt1-Y	3.219	0.52	0.10	8	4.19
Y-Si1	3.265	0.92	0.18	16	14.66
Pt2-Y	3.397	0.40	0.09	16	6.44
Pt1-Y	3.412	0.34	0.09	8	2.68
Pt1-Pt1	3.743	0.04	0.02	8	0.32
Y-Y	4.061	0.24	0.07	8	1.93
Y$_2$**Cu**$_3$Si$_5$ *tP*40					
Si2-Si2	2.369	3.73	0.62	2	7.46
Cu1-Si3	2.384	1.16	0.19	8	9.27
Cu2-Si3	2.390	1.09	0.16	16	17.45
Cu1-Si2	2.466	1.01	0.15	16	16.12
Si1-Si3	2.486	2.90	0.52	16	46.37

Cu1-Si3	2.521	0.68	0.10	8	5.44
Cu1-Si1	2.539	0.65	0.10	16	10.34
Cu1-Cu1	2.684	0.15	0.03	8	1.18
Cu2-Cu2	2.759	0.12	0.02	4	0.47
Si1-Si1	2.759	1.68	0.30	8	13.42
Cu2-Si1	2.786	0.31	0.05	8	2.44
Y-Si3	2.889	2.12	0.38	8	16.97
Y-Si3	2.937	1.77	0.32	16	28.36
Y-Si2	2.968	1.63	0.33	16	26.02
Y-Si1	3.039	1.57	0.31	16	25.13
Y-Cu1	3.092	0.41	0.07	16	6.56
Y-Si1	3.135	1.12	0.21	16	17.94
Y-Cu2	3.142	0.33	0.06	16	5.33
Si2-Si2	3.149	0.76	0.13	2	1.53
Y-Cu1	3.533	0.13	0.03	16	2.01
Y-Cu1	3.695	0.07	0.02	8	0.57
Si2-Si3	3.742	0.14	0.03	16	2.21
Y-Y	3.849	0.36	0.08	8	2.85
Y_2**Cu**$_3Si_5$ *oI*40					
Cu1-Si3	2.275	1.41	0.21	8	11.31
Cu1-Si1	2.358	1.03	0.15	16	16.51
Cu2-Si3	2.390	1.08	0.16	16	17.31
Cu1-Si2	2.397	0.94	0.14	16	15.00
Si1-Si3	2.436	3.16	0.54	16	50.55
Cu2-Si1	2.707	0.37	0.06	8	2.93
Cu2-Cu2	2.843	0.09	0.02	4	0.35
Si1-Si1	2.843	1.23	0.25	8	9.82
Si2-Si2	2.843	1.64	0.30	4	6.54
Si1-Si2	2.916	1.29	0.22	8	10.31
Y-Si3	2.930	2.07	0.38	8	16.54
Cu1-Y	3.022	0.48	0.08	8	3.83
Y-Si3	3.053	1.38	0.26	16	22.12
Cu1-Y	3.071	0.45	0.08	16	7.13
Y-Si3	3.073	1.27	0.25	8	10.12
Y-Si2	3.109	1.31	0.27	16	20.99
Cu1-Y	3.112	0.34	0.07	8	2.75
Y-Si1	3.164	1.00	0.18	16	15.92
Y-Si1	3.170	1.05	0.21	16	16.76
Cu2-Y	3.208	0.32	0.07	16	5.08
Cu1-Y	3.613	0.08	0.02	8	0.62
Cu1-Cu1	3.755	0.03	0.01	8	0.22
Y-Y	3.989	0.27	0.08	8	2.15

Multicentre interactions – ICOBI$^{(3)}$

Table S5. List of the three centre interactions and their related ICOBI$^{(3)}$ for the $Y_2M_3Si_5$(M= Fe, Co, Ni) in the tP40-$Sc_2Fe_3Si_5$, mS40-$Lu_2Co_3Si_5$ and oI40-$U_2Co_3Si_5$ type structure, respectively.

**$Y_2Fe_3Si_5$**	
Interactions	*ICOBI$^{(3)}$*
Si1-Fe1-Si1	0.000
Si2-Fe1-Si2	0.026
Si1-Fe1-Si3	0.011
Fe2-Si3-Fe2	–0.016
Fe1-Si3-Fe1	–0.005
Fe1-Si1-Fe1	–0.010
Fe1-Si3-Y	0.045
Fe2-Si3-Y	0.040
Fe1-Si3-Y	0.038
Si1-Si3-Si1	0.041
Si3-Si1-Si3	–0.012
Si1-Si1-Si1	–0.025
Si2-Si2-Si2	0.000
Fe2-Fe2-Fe2	–0.028
Si3-Y-Si3	–0.016
Y-Si3-Y	–0.007
Fe2-Si3-Fe1	–0.030

**$Y_2Co_3Si_5$**	
Interactions	*ICOBI$^{(3)}$*
Si1-Co1-Si4	0.007
Si2-Co1-Si2	–0.000
Si1-Co1-Si3	–0.029
Si4-Co1-Si2	0.000
Co2-Si3-Co2	–0.004
Co1-Si2-Co1	–0.019
Co1-Si2-Co1	–0.012
Co1-Si3-Y	0.024
Co1-Si3-Y	0.027
Co1-Si3-Y	0.031
Si1-Si3-Si4	0.046
Si3-Si4-Si3	-0.014
Si4-Si1-Si4	-0.031
Si2-Si2-Si2	-0.019
Co2-Co2-Co2	-0.014
Si3-Y-Si3	-0.005
Si3-Y- Si3	-0.016

**$Y_2Ni_3Si_5$**	
Interactions	*ICOBI$^{(3)}$*
Si1-Ni1-Si1	0.000
Si2-Ni1-Si2	–0.006
Si1-Ni1-Si3	0.003
Ni2-Si3-Ni2	0.004
Ni1-Si2-Ni1	–0.011
Ni1-Si2-Ni1	–0.009
Ni1-Si3-Y	0.029
Ni1-Si3-Y	0.022
Ni1-Si3-Y	0.023
Si1-Si3-Si1	0.044
Si1-Si1-Si1	–0.021
Si2-Si2-Si2	–0.028
Ni2-Ni2-Ni2	–0.026
Si3-Y- Si3	–0.000
Y- Si3- Y	–0.026
Si3-Y-Si3	–0.007
Ni2-Si3-Ni1	–0.013
Si3-Ni1-Si1	–0.001
Y- Si3- Y	0.001
Ni1-Si1-Si3	–0.001

Periodic Trends

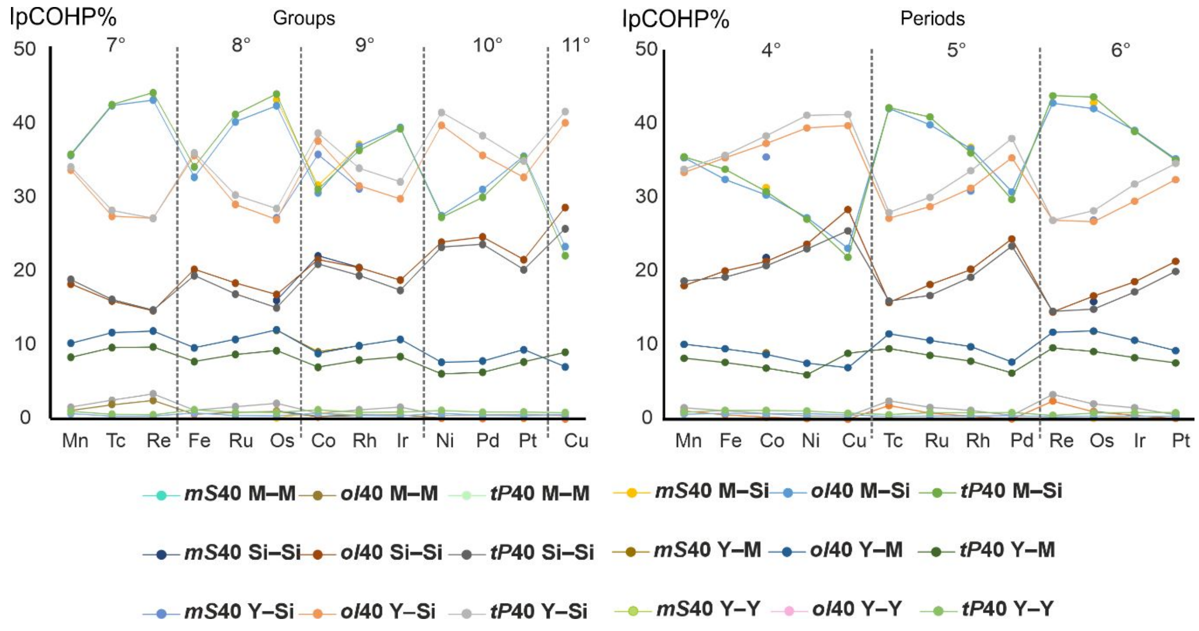

Figure S2. IpCOHP% vs Transition Metal (M) plots. The M elements are ordered by group (left) and by period (right). Each colour corresponds to a specific interaction within a given structure, as indicated in the legend. To highlight trends, lines are included as a guide to the eye.

Y–Si interactions in $Y_2M_3Si_5$

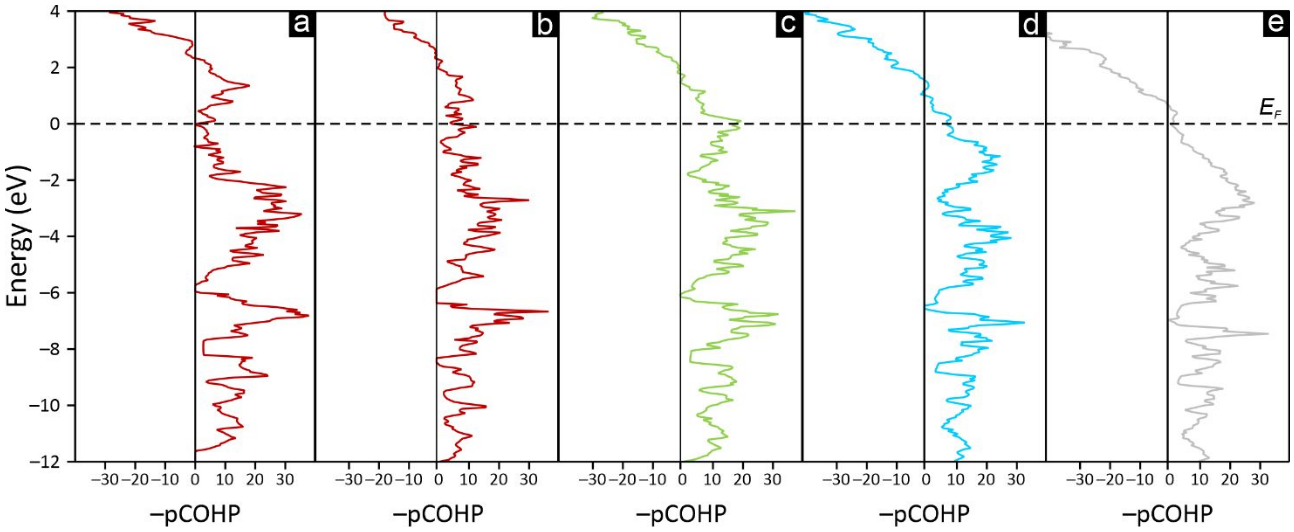

Figure S3. Y-Si pCOHP curve for the $Y_2M_3Si_5$ compound, a)Mn, b)Fe, c)Co, d)Ni, e)Cu.
